# Supplementary material for: Path entropy-driven design of solid-state electrolytes
Source: Nat Commun. 2026 Apr 1;17:4736. doi: 10.1038/s41467-026-71316-z (PMC13216346; doi:10.1038/s41467-026-71316-z)
Supplement: Supplementary file 1 — Supplementary Information [file 41467_2026_71316_MOESM1_ESM.pdf]

Supplementary Information

Path Entropy-driven Design of Solid-State Electrolytes

Qiye Guan[a,*], Kaiyang Wang[b], Jingjie Yeo[b], Yongqing Cai[a,*]

[a] Institute of Applied Physics and Materials Engineering, University of Macau, Taipa, Macau, China

[b] Department of Materials Science and Engineering, Cornell University, Ithaca, New York 14853, United States

[*]Corresponding authors: qiye.guan@connect.um.edu.mo; yongqingcai@um.edu.mo

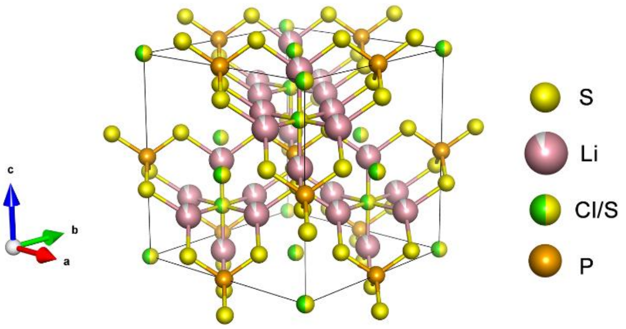

Supplementary Figure 1. Site positions of experimentally validated LPSCl-III ($Li_{5.5}PS_{4.5}Cl_{1.5}$)[1].

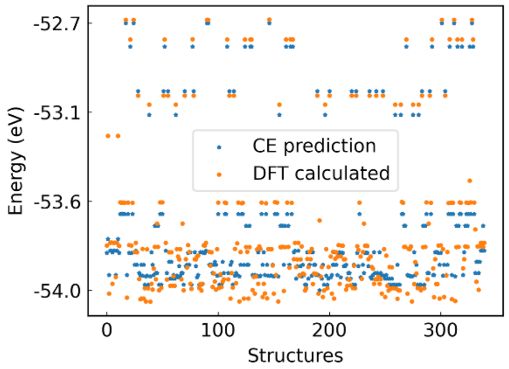

Supplementary Figure 2. Construction of the cluster expansion model of LPSCl-III. By calculating 340 initial structures, we construct our cluster expansion (CE) model through the Statistical Mechanics on Lattices (smol) package[2]. For all these structures, first-principles calculations are performed through the Vienna ab initio simulation package (VASP)[3] with the projector augmented wave (PAW) method. The exchange-correlation energy is computed within the GGA, employing the PBE functional[4]. Additionally, the DFT-D3 functional with Grimme correction is utilized to account for weak vdW interactions[5]. The calculations are conducted with an energy cutoff of 450 eV and an energy convergence criterion of 10^{-4} eV.

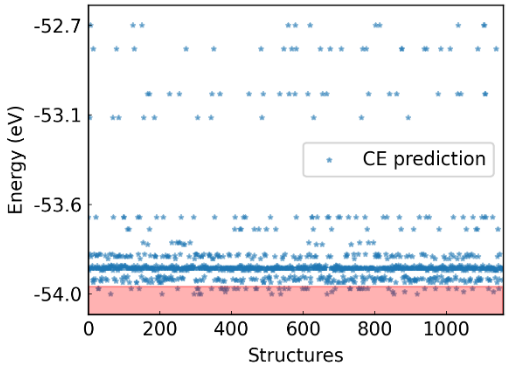

Supplementary Figure 3. Selection of LPSCl-III candidates. The red area shows the possible stable candidates.

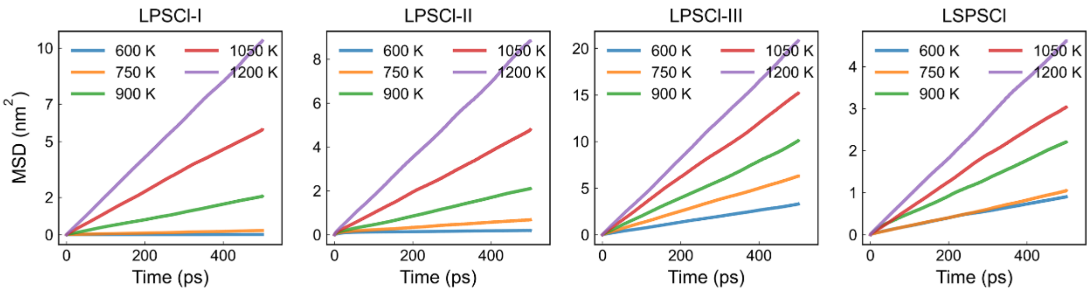

Supplementary Figure 4. Mean squared displacements (MSDs) of all four argyrodite-type SSEs.

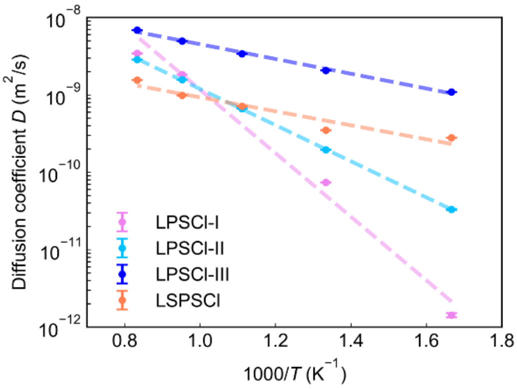

Supplementary Figure 5. Diffusion coefficients (D) as a function of temperature for four argyrodite-type SSEs. The uncertainty was estimated using block averaging with

N = 5 independent time origins. Data are presented as mean values, with error bars denoting the 95% confidence interval.

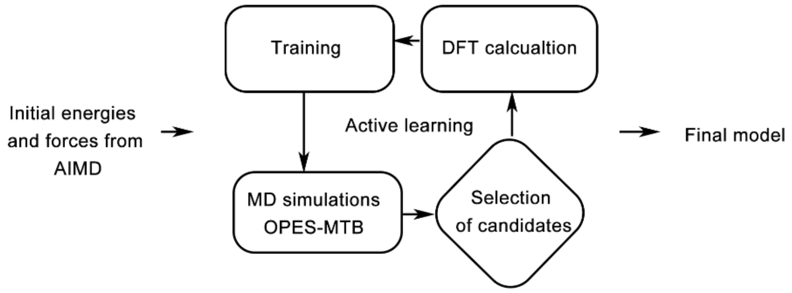

Supplementary Figure 6. Workflow in training neural-network potentials (NNPs)**.** Active learning was employed for MLP training, utilizing the dp-gen package[6].

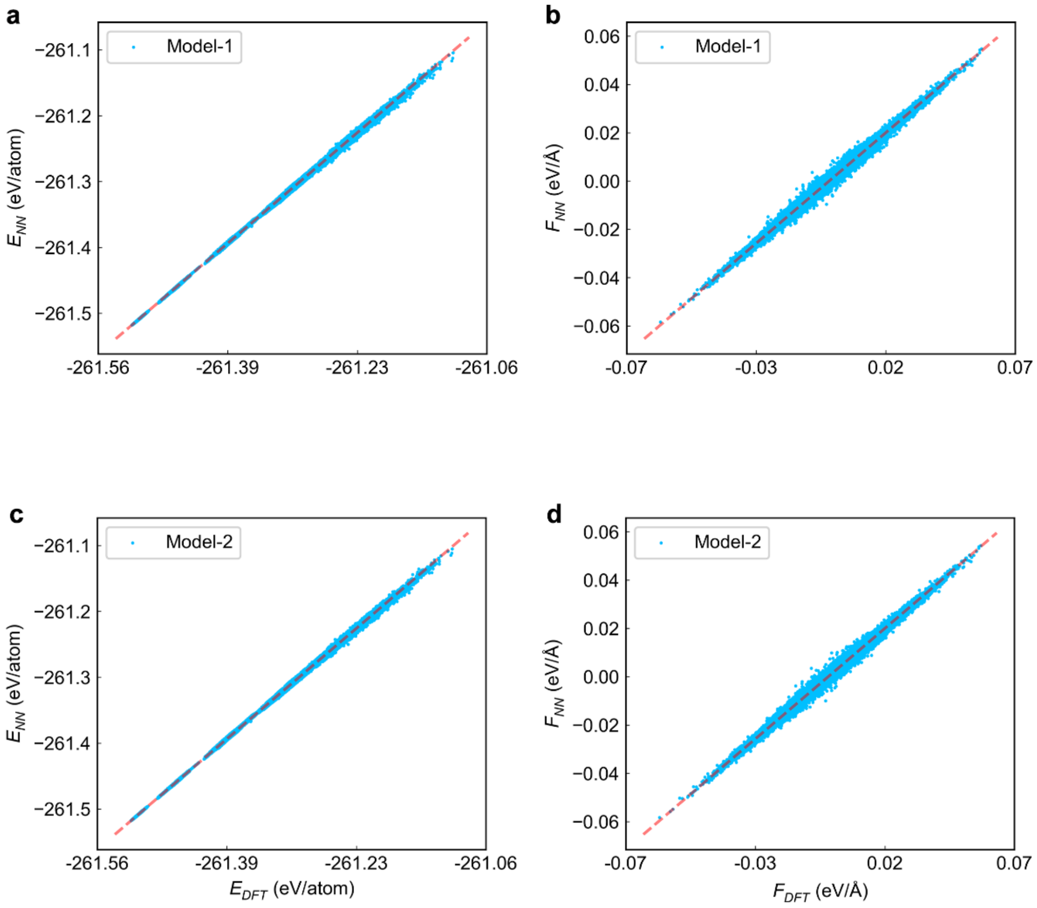

Supplementary Figure 7. Evaluation of NNPs of LPSCl-I. a-b, The differences of energies and forces between DFT calculation and NNP from model-1, respectively. **c-**

d, The differences of energies and forces between DFT calculation and NNP from model-2, respectively. The red dotted line represents the energy/force calculated from DFT.

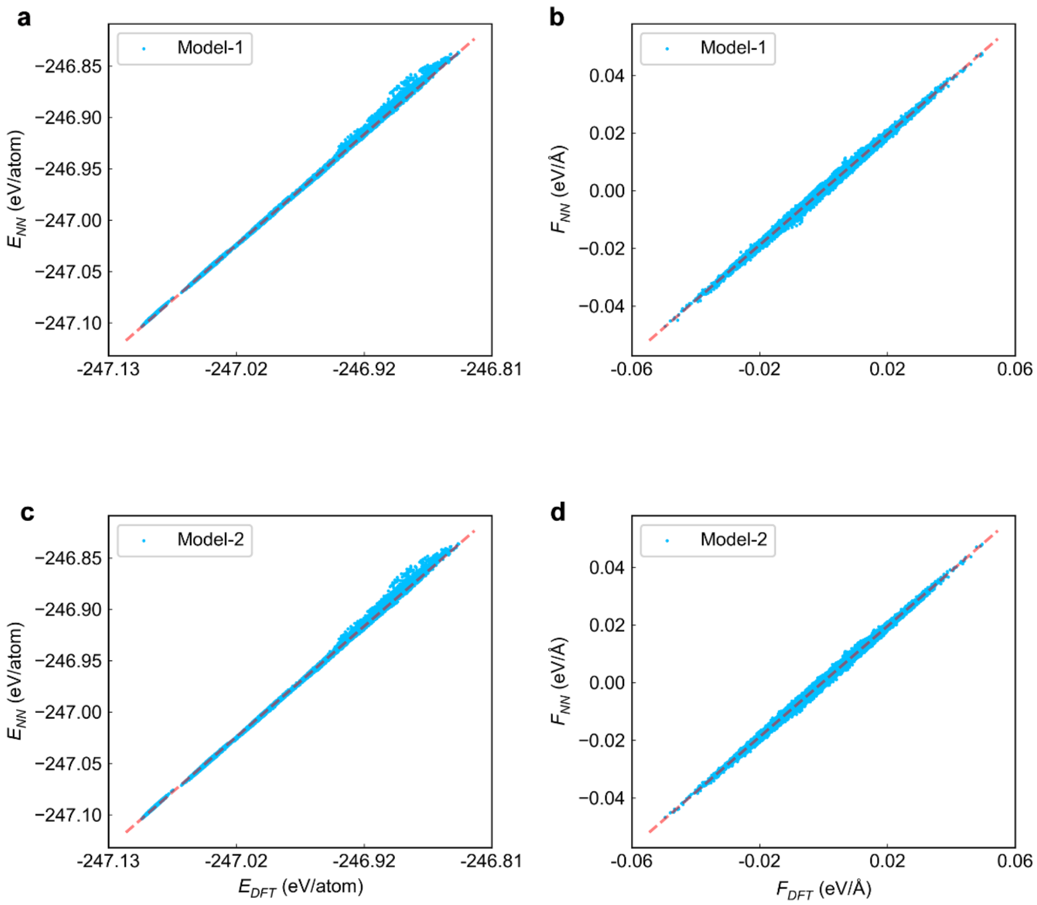

Supplementary Figure 8. Evaluation of NNPs of LPSCl-II. a-b, The differences of energies and forces between DFT calculation and NNP from model-1 and model-2, respectively. **c-d,** The differences of energies and forces between DFT calculation and NNP from model-2, respectively. The red dotted line represents the energy/force calculated from DFT.

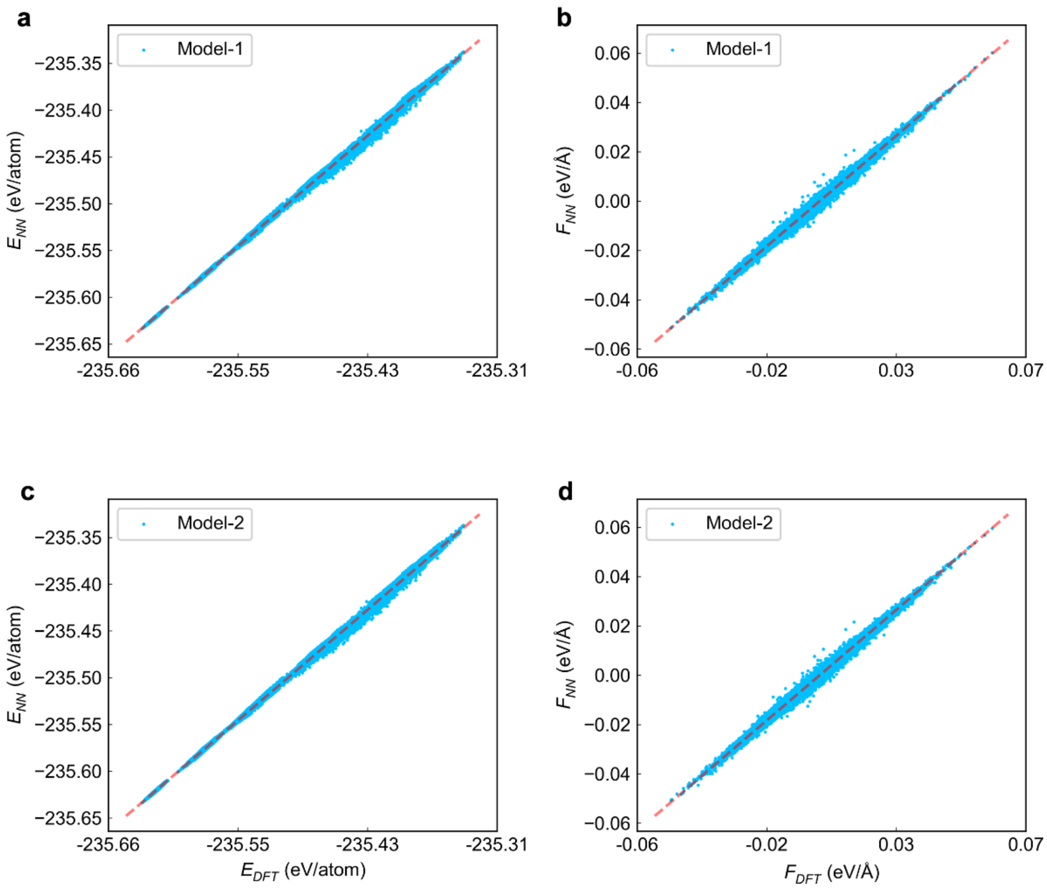

Supplementary Figure 9. Evaluation of NNPs of LPSCl-III. a-b, The differences of energies and forces between DFT calculation and NNP from model-1 and model-2, respectively. **c-d,** The differences of energies and forces between DFT calculation and NNP from model-2, respectively. The red dotted line represents the energy/force calculated from DFT.

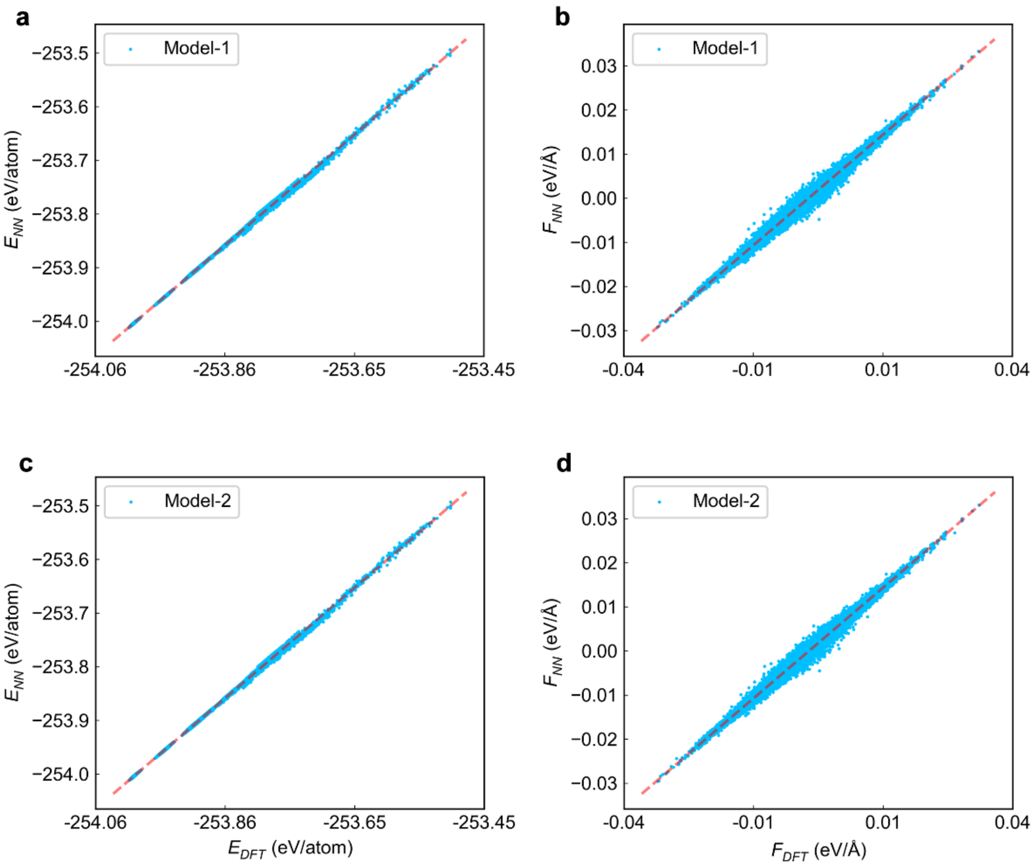

Supplementary Figure 10. Evaluation of NNPs of LSPSCl. a-b, The differences of energies and forces between DFT calculation and NNP from model-1, respectively. **c-d,** The differences of energies and forces between DFT calculation and NNP from model-2, respectively. The red dotted line represents the energy/force calculated from DFT.

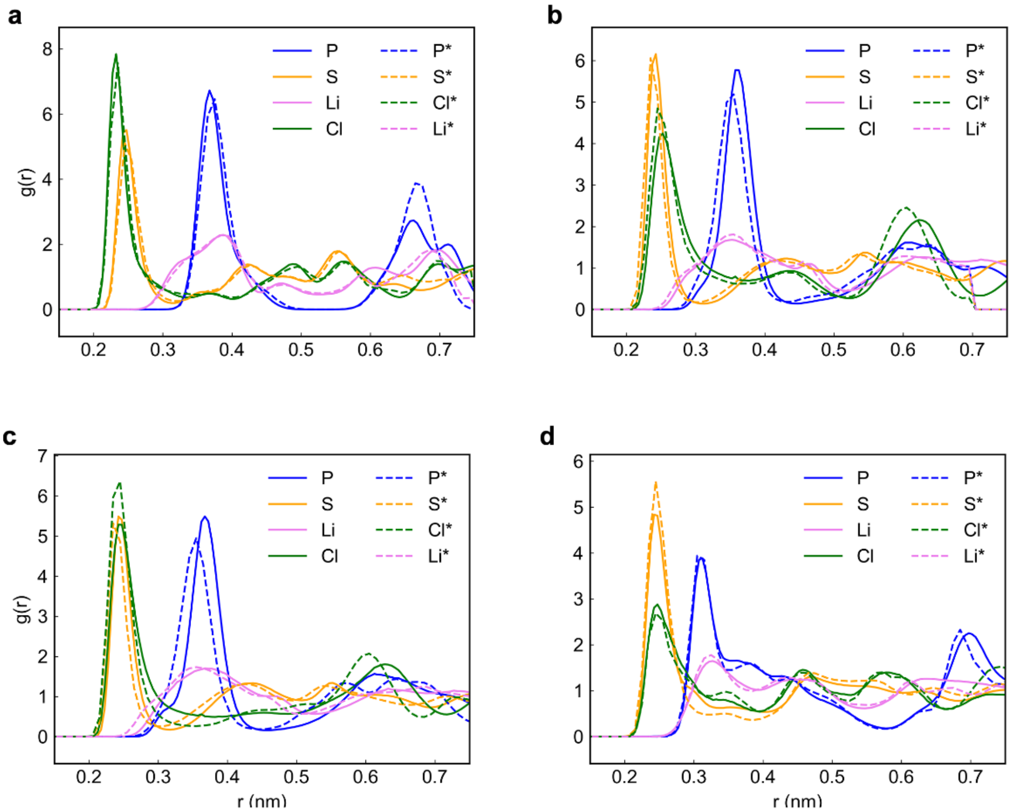

Supplementary Figure 11. Radial distribution function plot of Li with P, S, Li, and Cl elements at 300 K under NVT ensemble. **a**, LPSCl-I, **b**, LPSCl-II, **c**, LSPSCl, and **d**, LPSCl-III. The dotted and solid lines represent the calculation from DFT and NNPs, respectively.

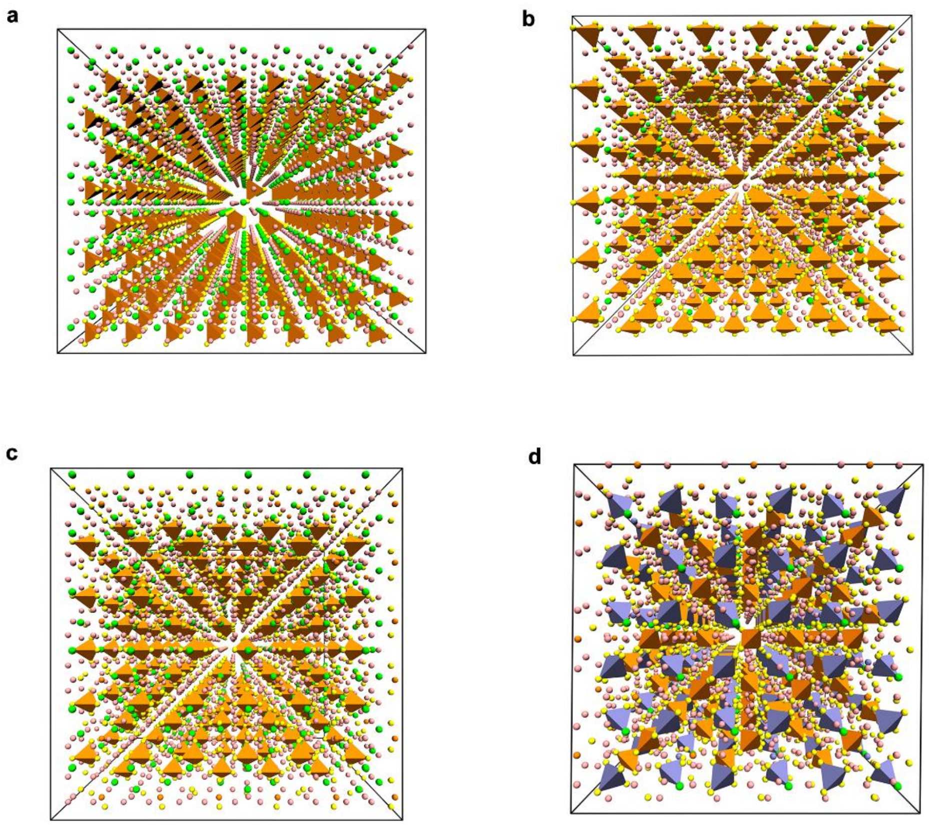

Supplementary Figure 12. Supercells of argyrodite-type SSEs for simulation. **a-d**, Superlattices of LPSCl-I (a), LPSCl-II (b), LPSCl-III (c), and LSPSCl (d). Li, P, S, and Cl atoms are colored pink, orange, yellow, and green, respectively. For LPSCl-I, a supercell with 4608 atoms (4.71 nm × 41.46 nm × 42.83 nm) was used. For LPSCl-II, a supercell with 3744 atoms (4.29 nm × 4.29nm × 4.04 nm) was used. For LPSCl-III, a supercell with 3600 atoms (4.36 nm × 4.36 nm × 4.11 nm) was used. For LSPSCl, a supercell with 3600 atoms (3.69 nm × 3.69 nm × 5.17 nm) was used.

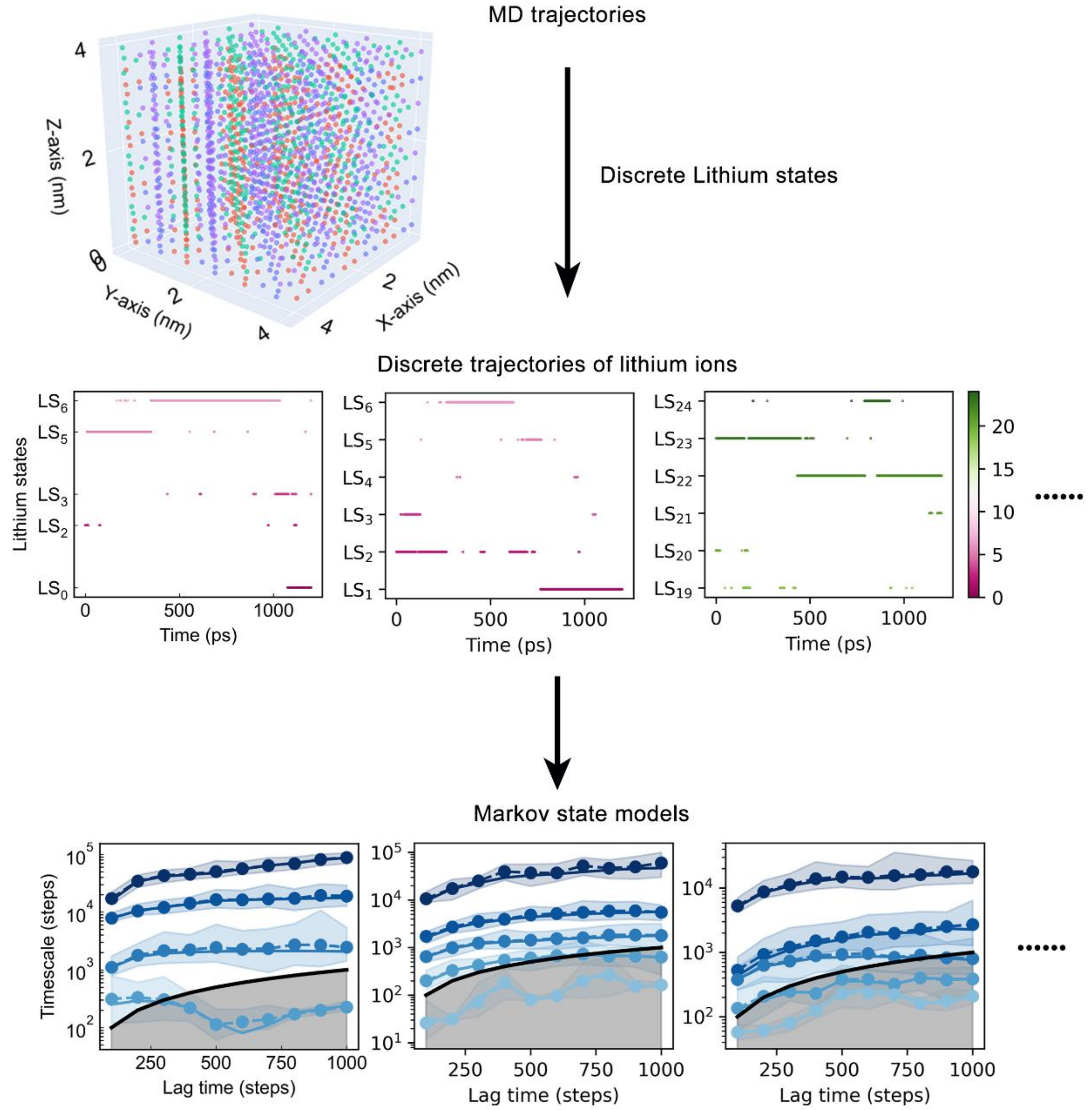

Supplementary Figure 13. Schematic diagram of MSM construction. MD simulations are performed under the NVT ensemble at 300 K through LAMMPS[7] with i-Pi[8] software. We then get lithium coordination shells (LCSs) through periodic K-means based clustering algorithm. The lithium ions in each LCS are then partitioned into subspace through a Voronoi partition procedure. Using discretized trajectories, the final MSM model is constructed through Deeptime package[9].

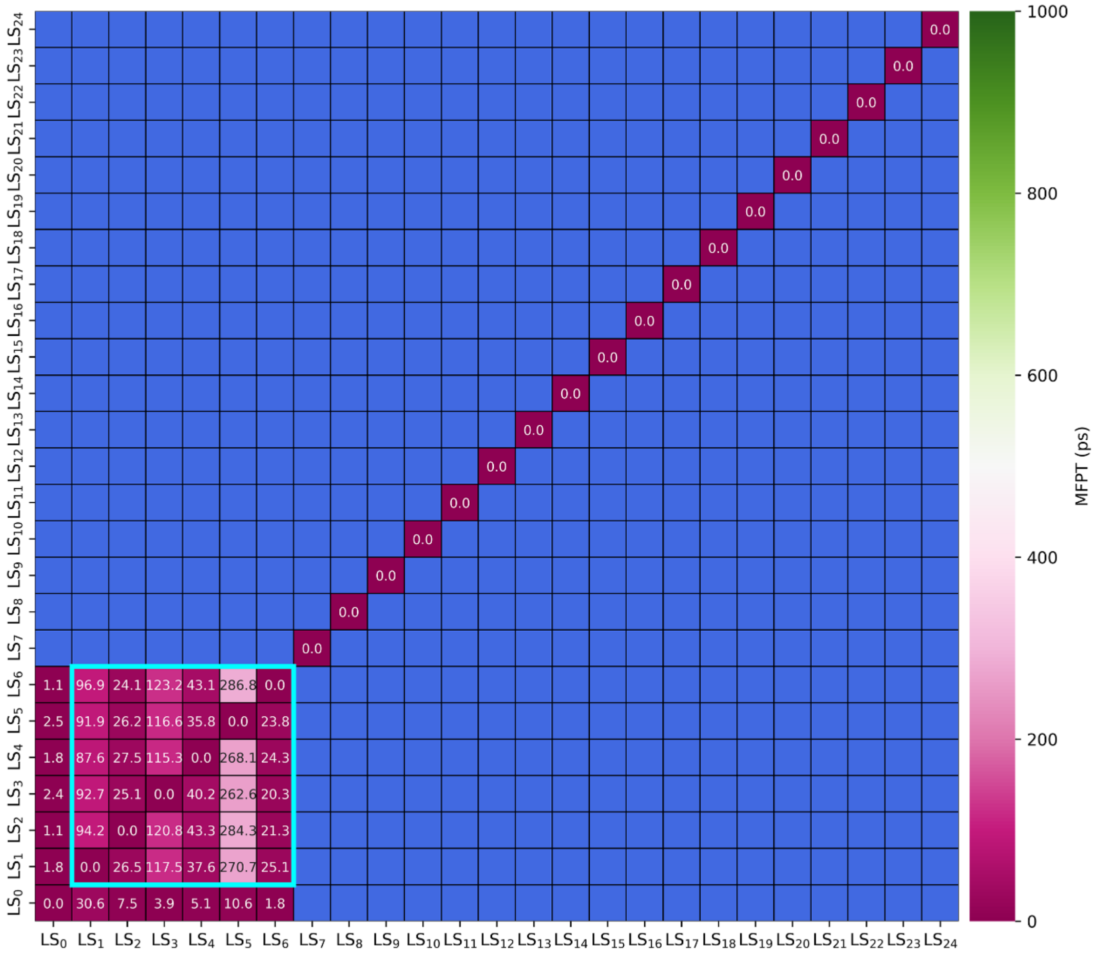

Supplementary Figure 14. MFPT profile of lithium hopping in LPSCl-II at 300 K of LCS-1. The initial lithium states in LCS-1 are denoted with a cyan rectangle. Blocks colored blue represent no transition happens or have MFPT value greater than 10000 ps.

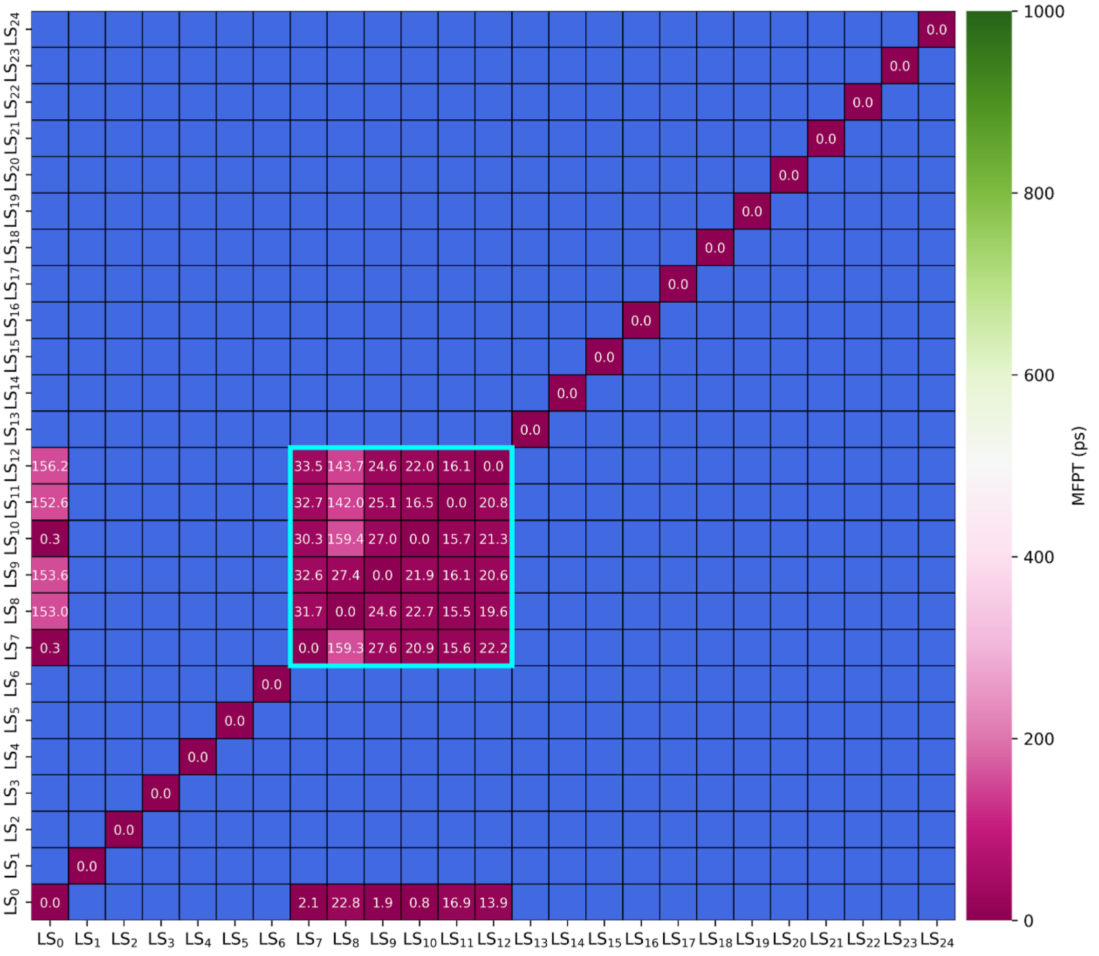

Supplementary Figure 15. MFPT profile of lithium hopping in LPSCl-II at 300 K of LCS-2. The initial lithium states in LCS-2 are denoted with a cyan rectangle. Blocks colored blue represent no transition happens or have MFPT value greater than 10000 ps.

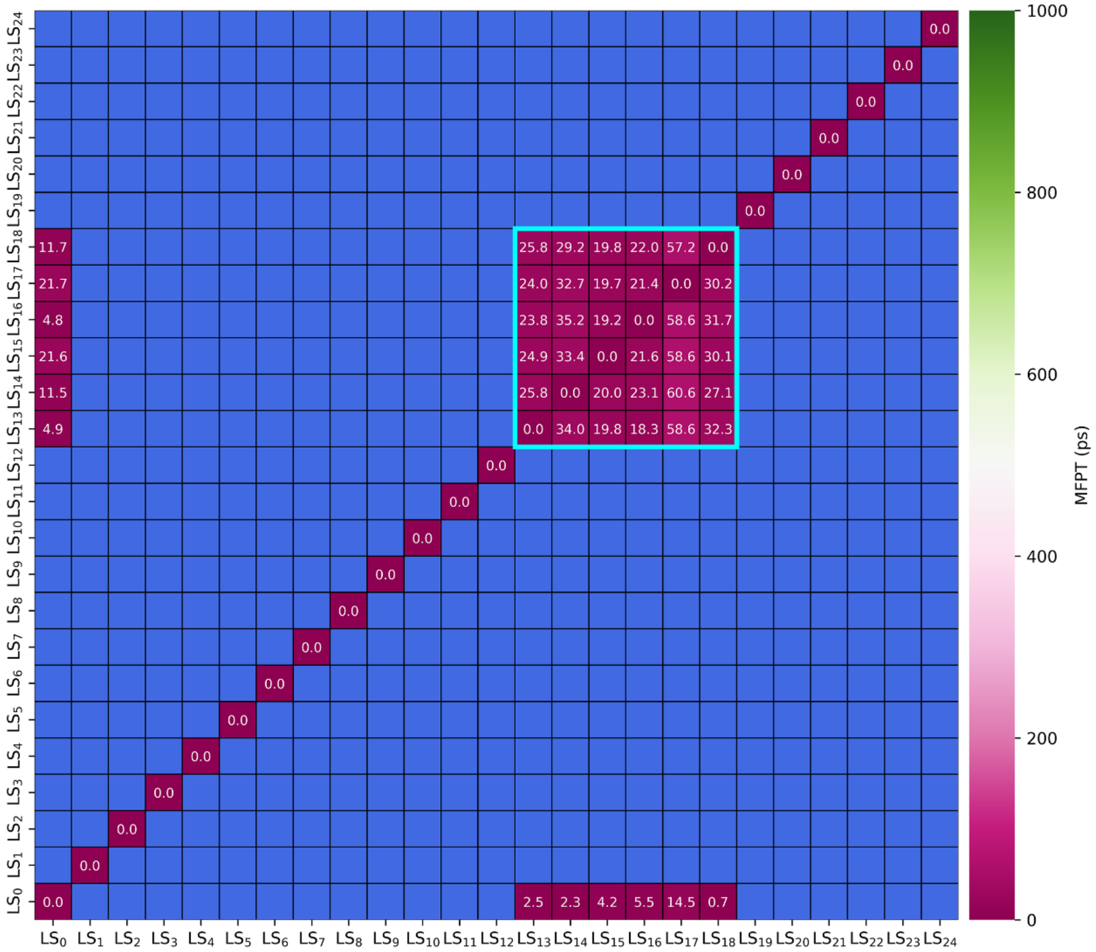

Supplementary Figure 16. MFPT profile of lithium hopping in LPSCl-II at 300 K of LCS-3. The initial lithium states in LCS-3 are denoted with a cyan rectangle. Blocks colored blue represent no transition happens or have MFPT value greater than 10000 ps.

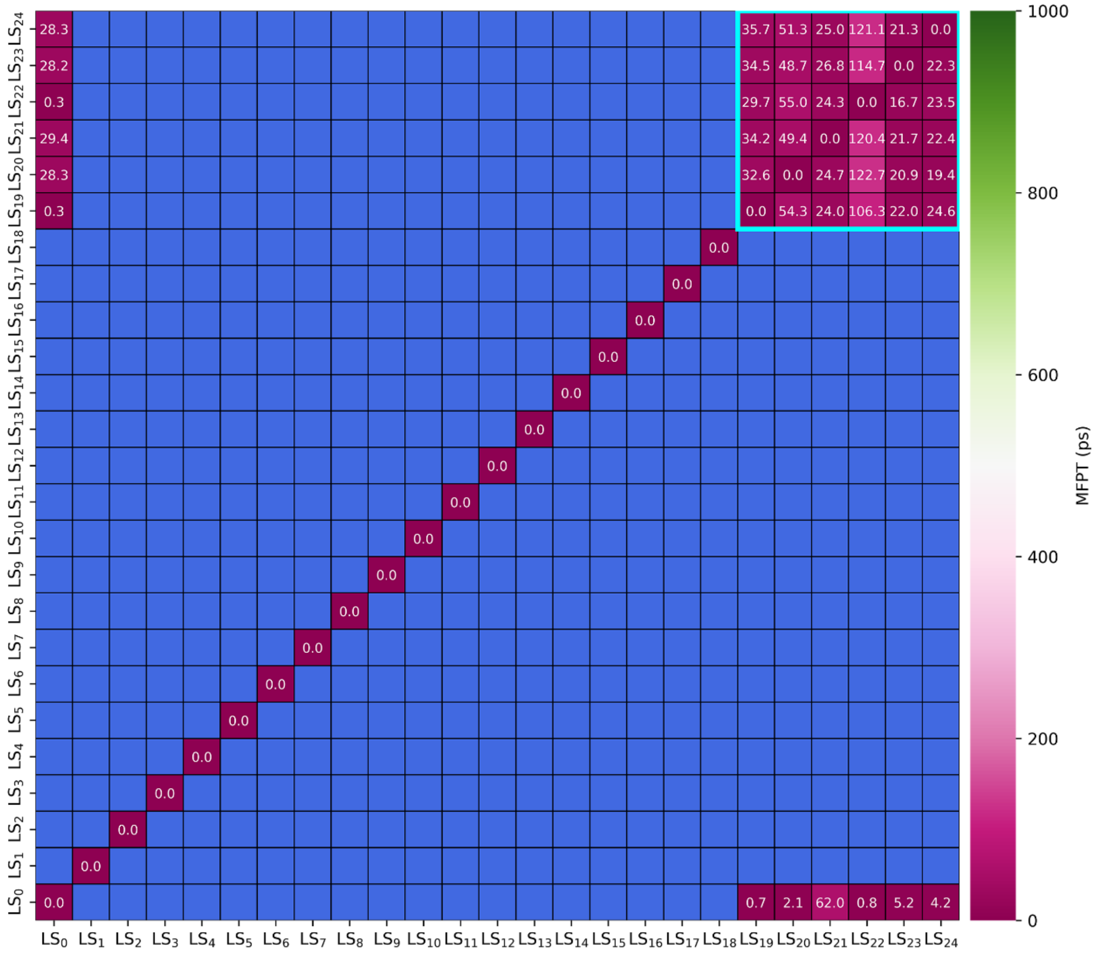

Supplementary Figure 17. MFPT profile of lithium hopping in LPSCl-II at 300 K of LCS-4. The initial lithium states in LCS-4 are denoted with a cyan rectangle. Blocks colored blue represent no transition happens or have MFPT value greater than 10000 ps.

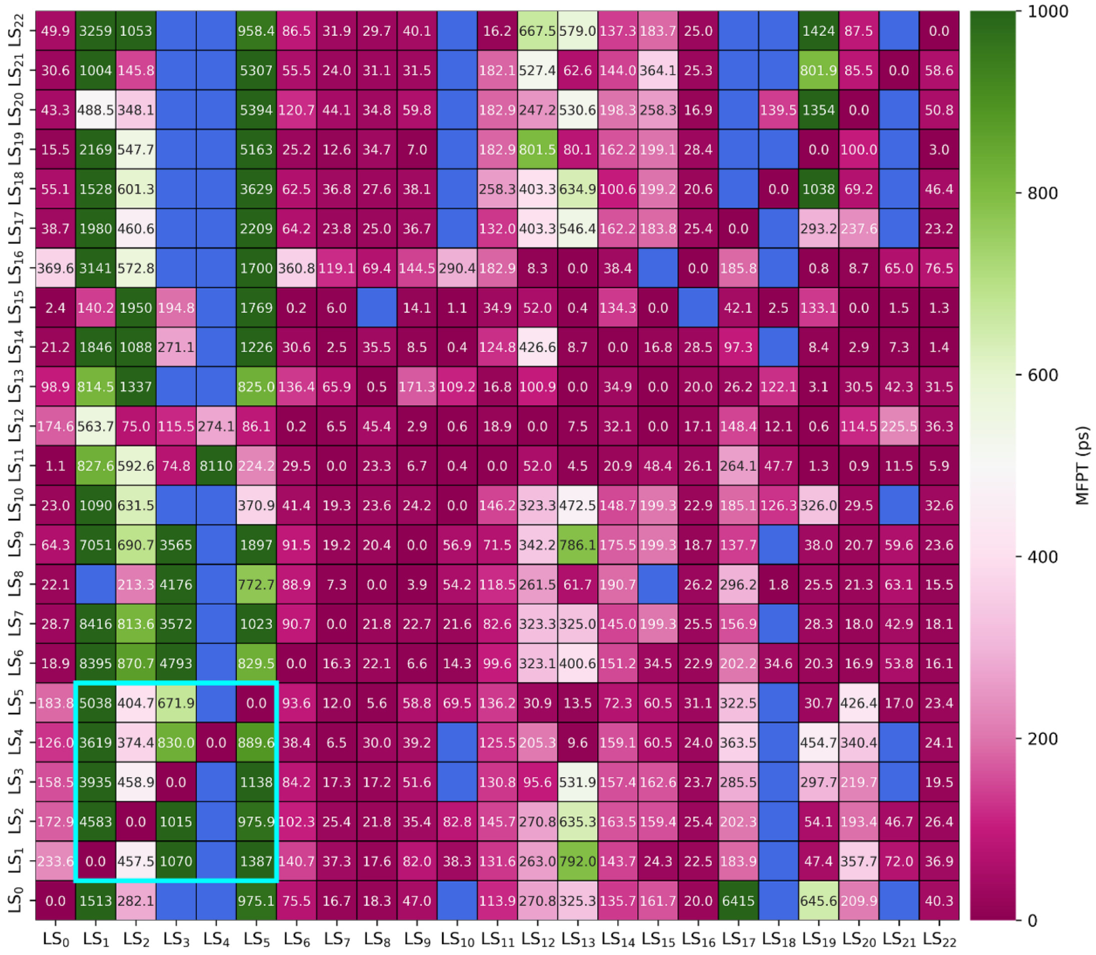

Supplementary Figure 18. MFPT profile of lithium hopping in LPSCl-III at 300 K of LCS-1. The initial lithium states in LCS-1 are denoted with a cyan rectangle. Blocks colored blue represent no transition happens or have MFPT value greater than 10000 ps.

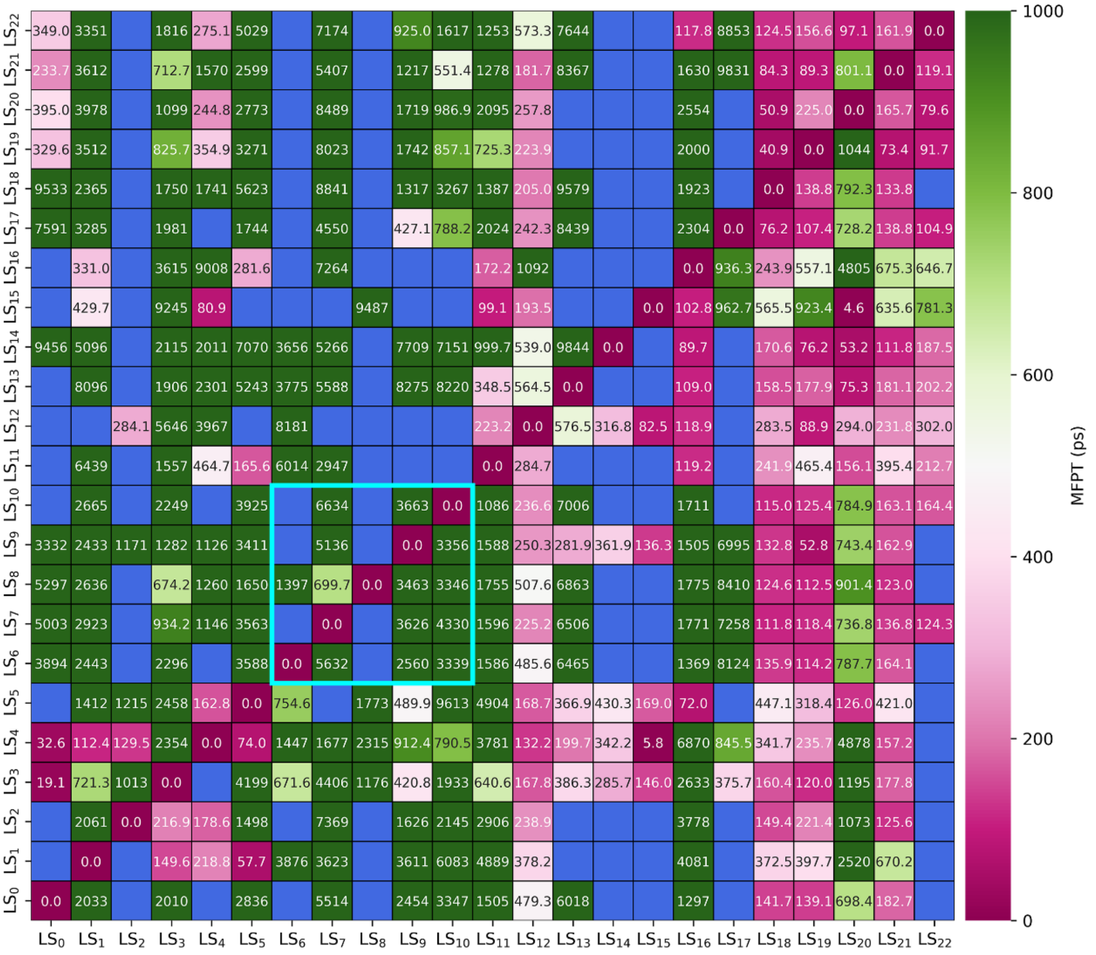

Supplementary Figure 19. MFPT profile of lithium hopping in LPSCl-III at 300 K of LCS-2. The initial lithium states in LCS-2 are denoted with a cyan rectangle. Blocks colored blue represent no transition happens or have MFPT value greater than 10000 ps.

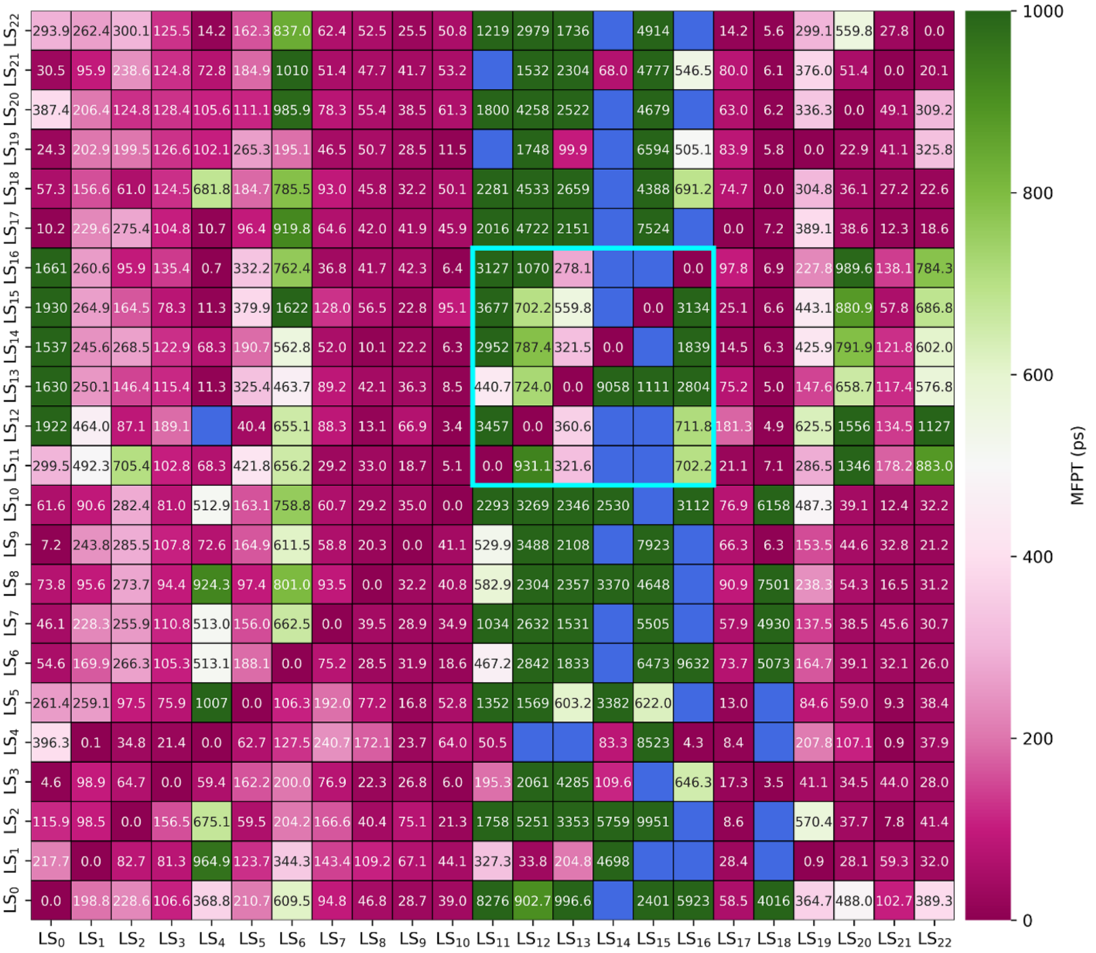

Supplementary Figure 20. MFPT profile of lithium hopping in LPSCl-III at 300 K of LCS-3. The initial lithium states in LCS-3 are denoted with a cyan rectangle. Blocks colored blue represent no transition happens or have MFPT value greater than 10000 ps.

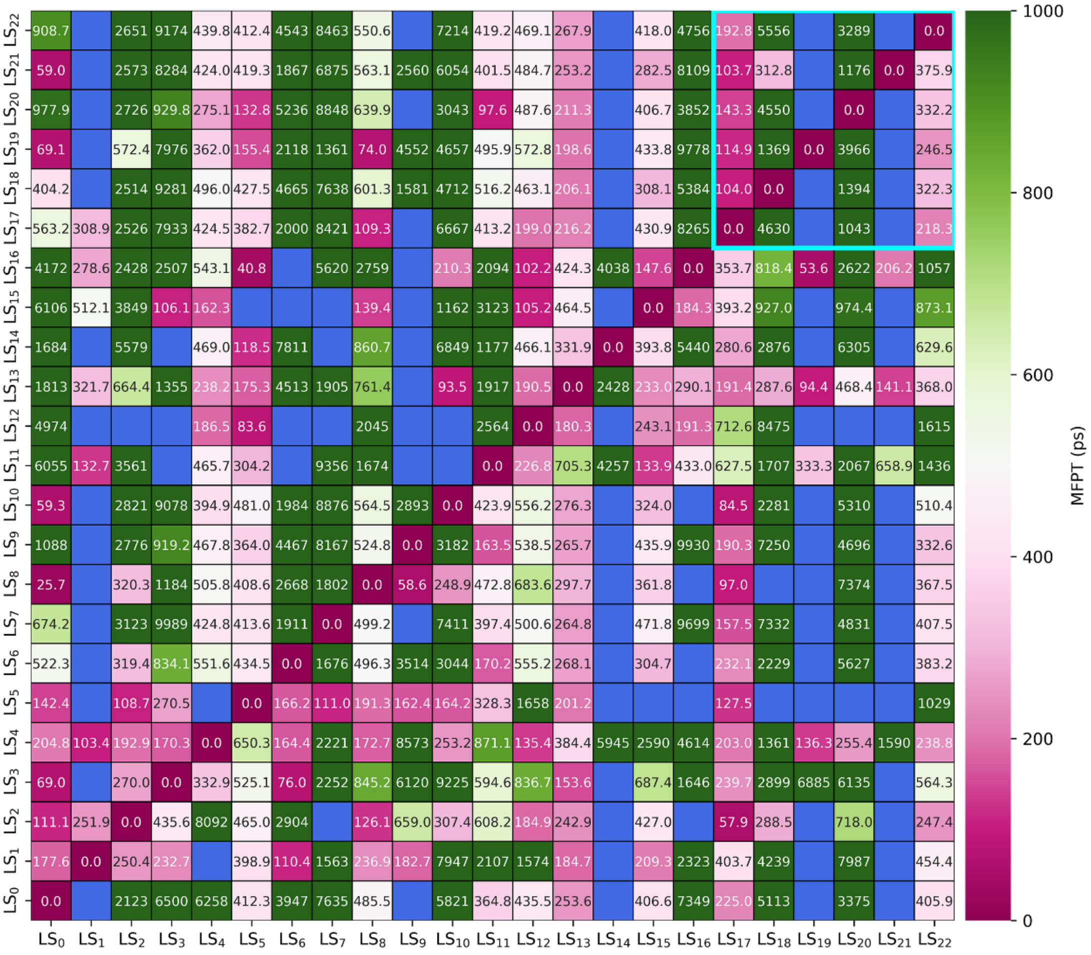

Supplementary Figure 21. MFPT profile of lithium hopping in LPSCl-III at 300 K of LCS-4. The initial lithium states in LCS-4 are denoted with a cyan rectangle. Blocks colored blue represent no transition happens or have MFPT value greater than 10000 ps.

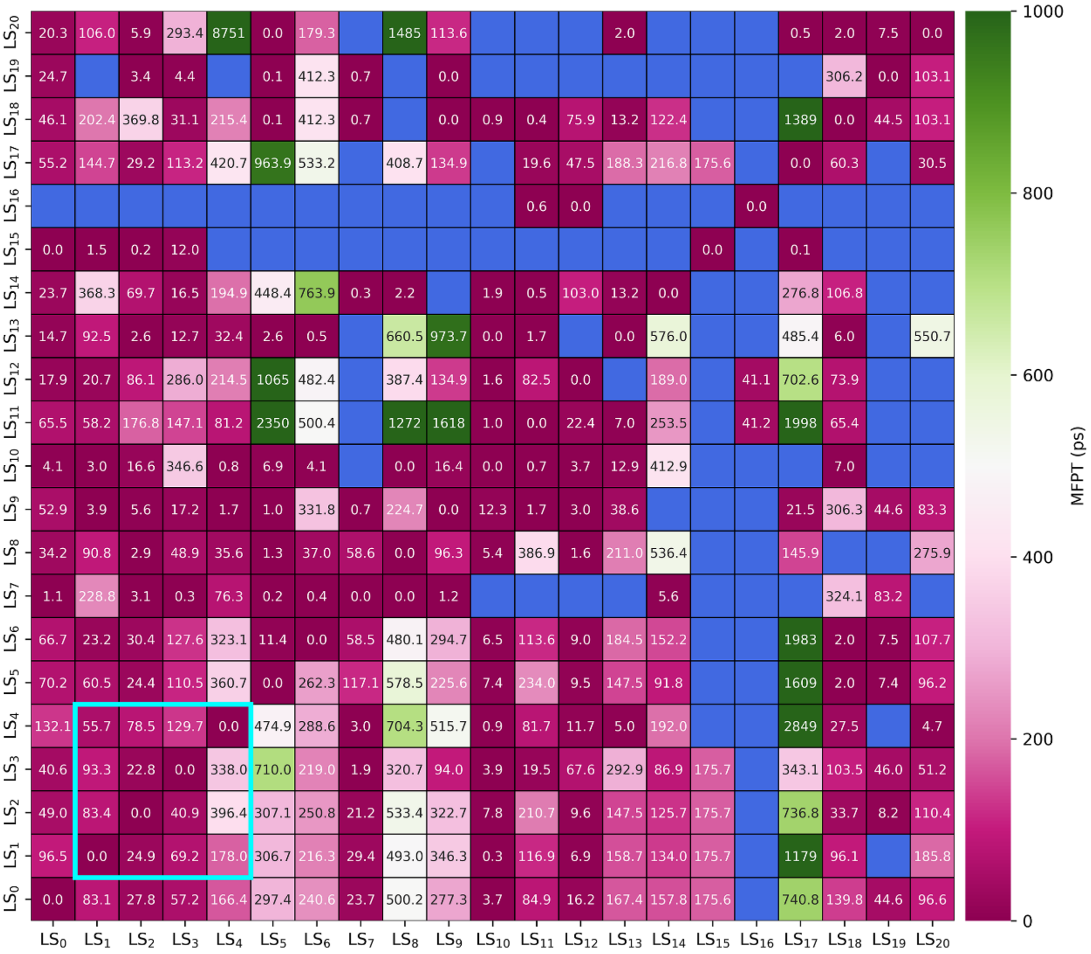

Supplementary Figure 22. MFPT profile of lithium hopping in LSPSCl at 300 K of LCS-1. The initial lithium states in LCS-1 are denoted with a cyan rectangle. Blocks colored blue represent no transition happens or have MFPT value greater than 10000 ps.

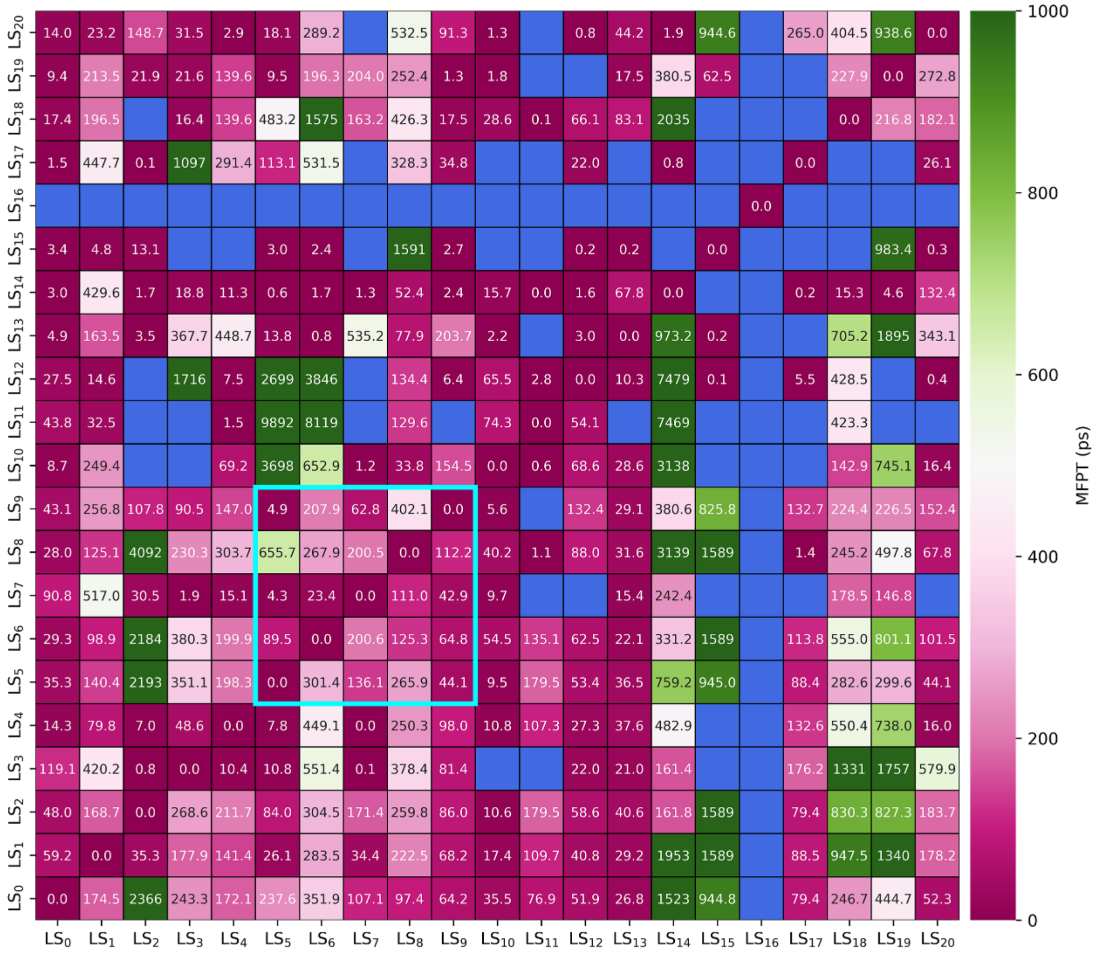

Supplementary Figure 23. MFPT profile of lithium hopping in LSPSCl at 300 K of LCS-2. The initial lithium states in LCS-2 are denoted with a cyan rectangle. Blocks colored blue represent no transition happens or have MFPT value greater than 10000 ps.

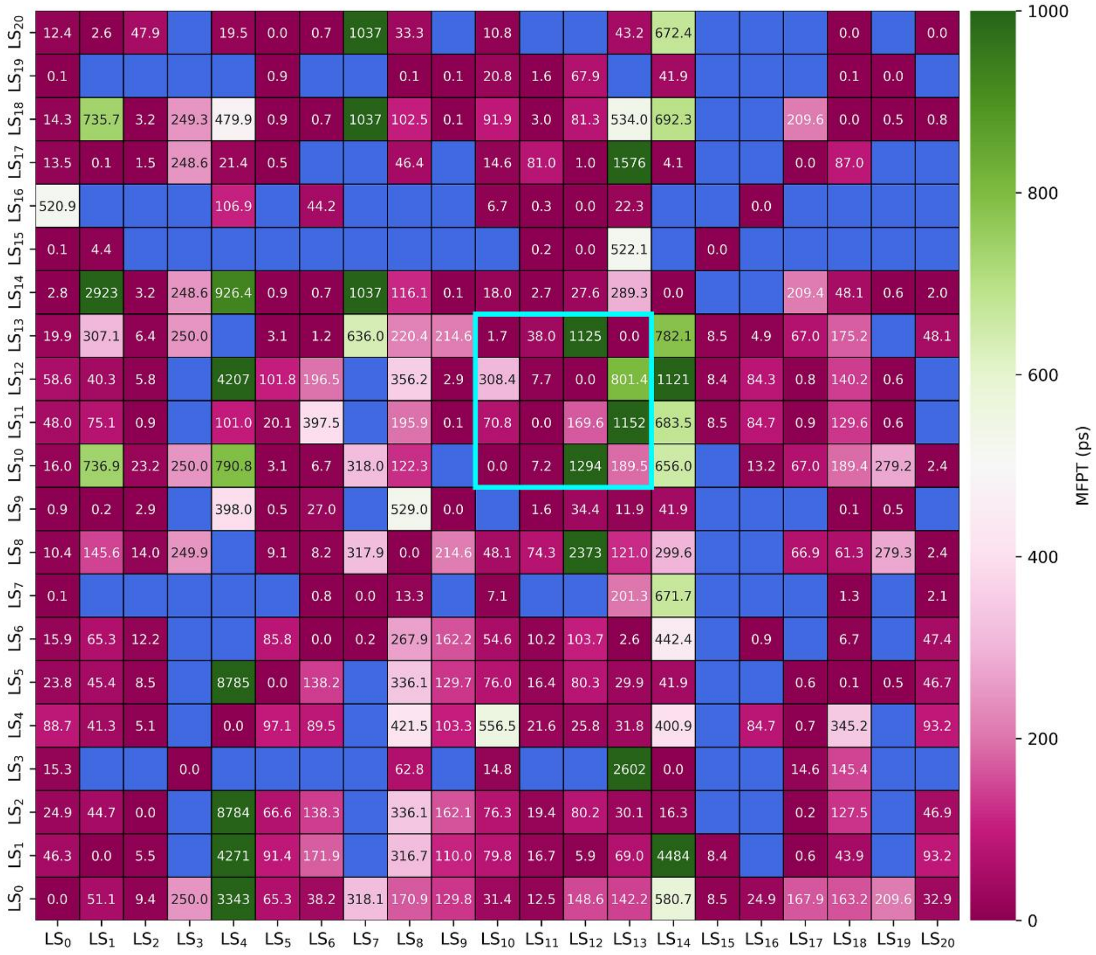

Supplementary Figure 24. MFPT profile of lithium hopping in LSPSCl at 300 K of LCS-3. The initial lithium states in LCS-3 are denoted with a cyan rectangle. Blocks colored blue represent no transition happens or have MFPT value greater than 10000 ps.

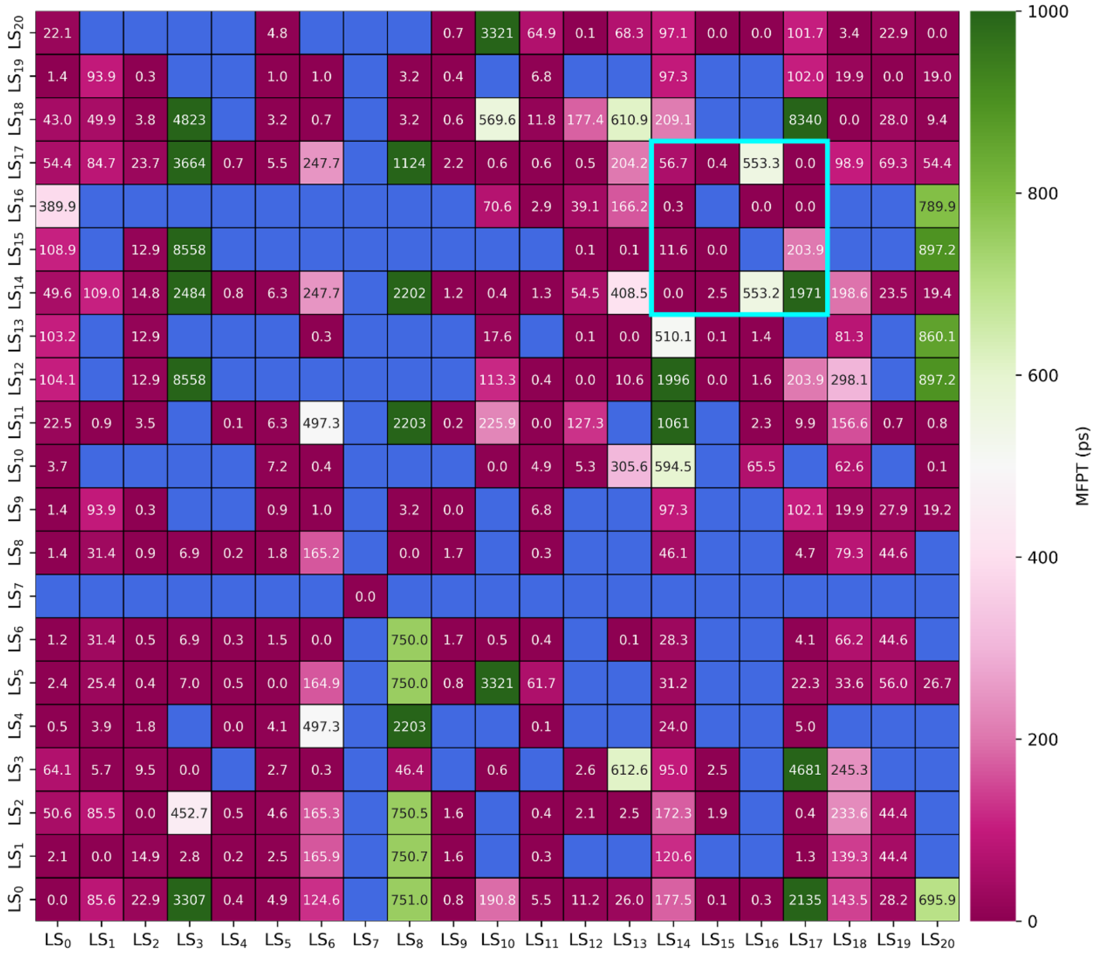

Supplementary Figure 25. MFPT profile of lithium hopping in LSPSCl at 300 K of LCS-4. The initial lithium states in LCS-4 are denoted with a cyan rectangle. Blocks colored blue represent no transition happens or have MFPT value greater than 10000 ps.

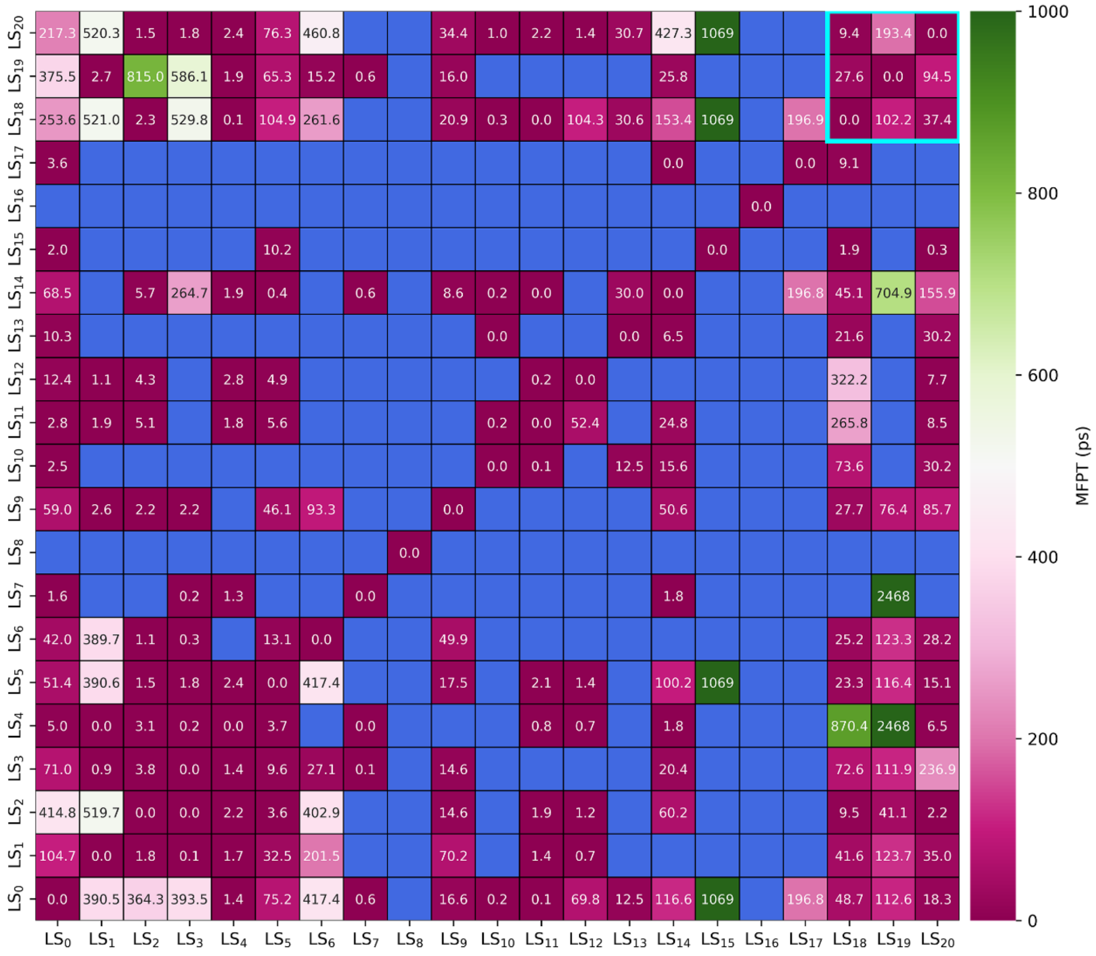

Supplementary Figure 26. MFPT profile of lithium hopping in LSPSCl at 300 K of LCS-5. The initial lithium states in LCS-5 are denoted with a cyan rectangle. Blocks colored blue represent no transition happens or have MFPT value greater than 10000 ps.

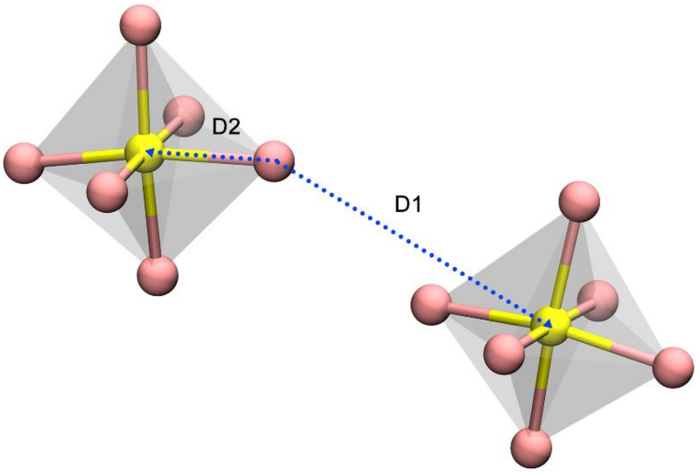

Supplementary Figure 27. Definition of the D1 and D2 in LPSCl-II and LPSCl-III.

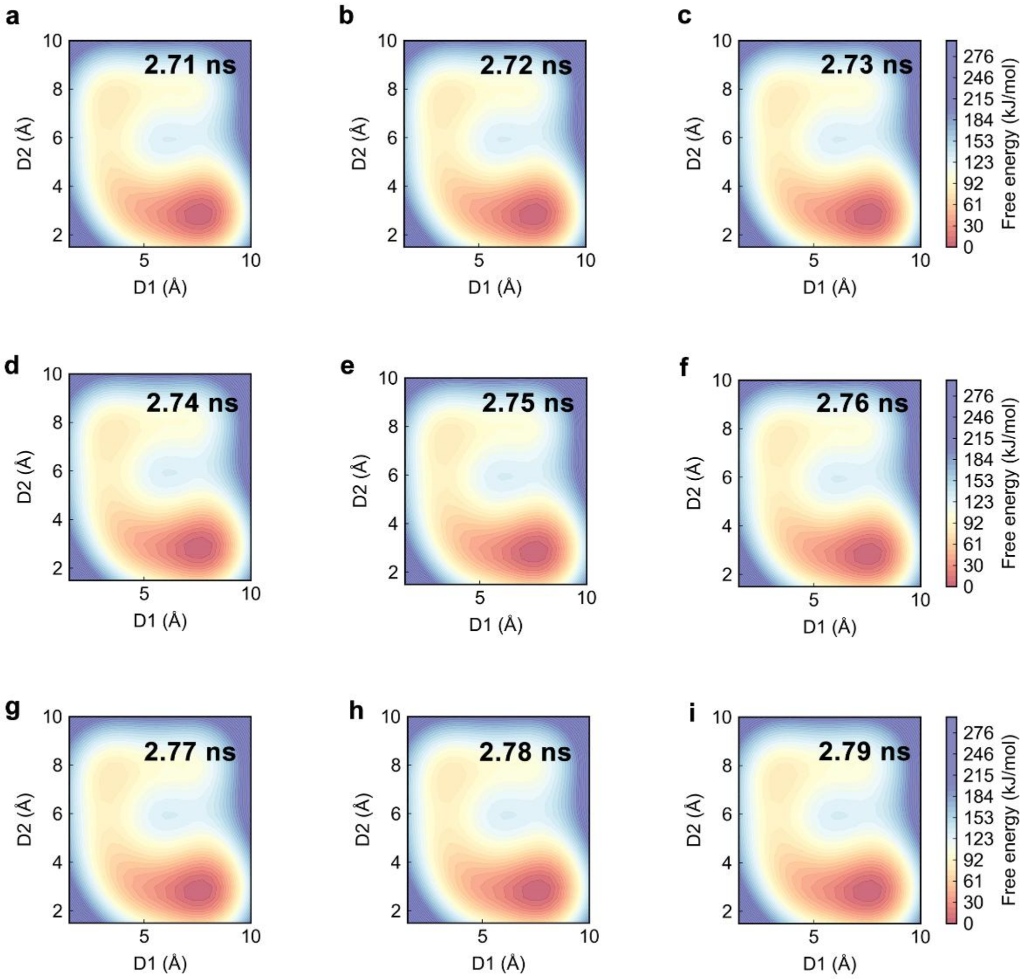

Supplementary Figure 28. Free energy convergence plot of inter-LCS lithium-ion diffusion of LPSCl-II from 2.71 ns to 2.79 ns.

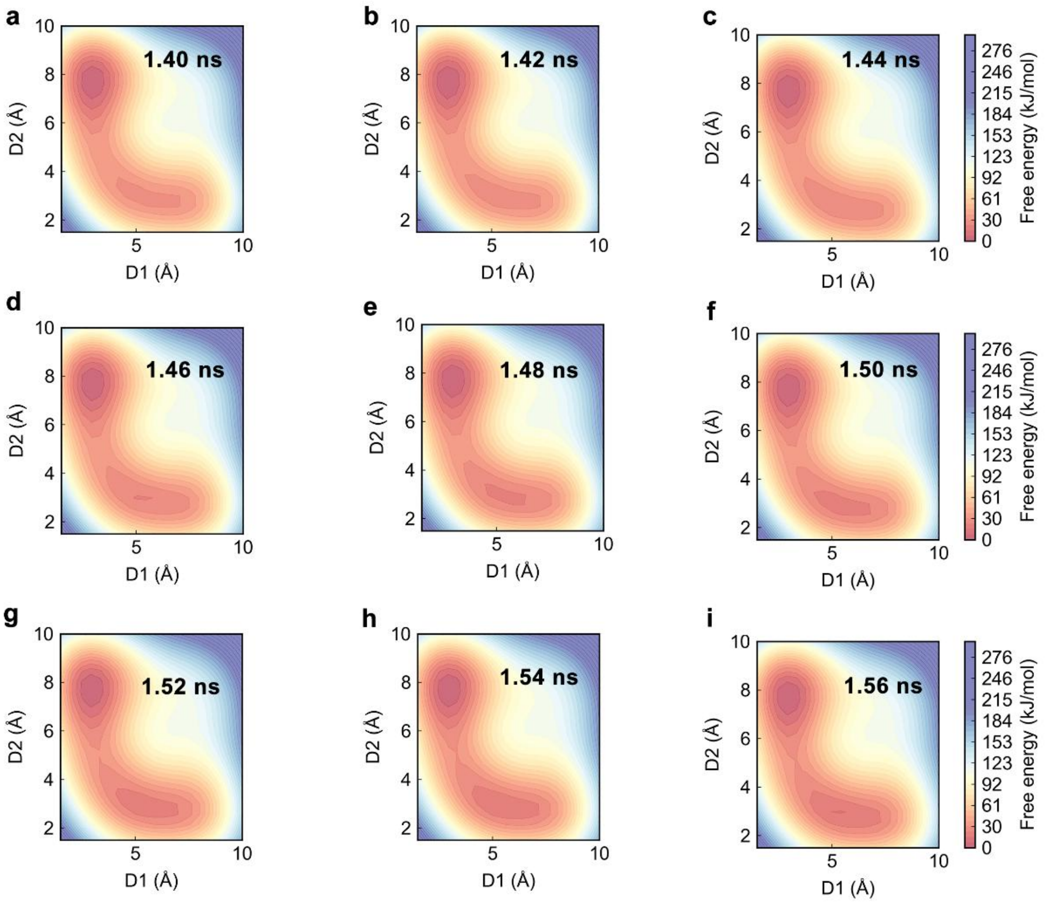

Supplementary Figure 29. Free energy convergence plot of inter-LCS lithium-ion diffusion of LPSCl-III from 1.40 ns to 1.56 ns.

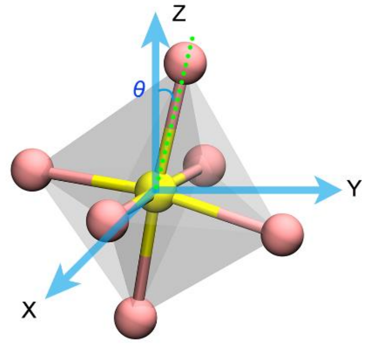

Supplementary Figure 30. Definition of the rotation angle θ in LPSCl-II and LPSCl-III.

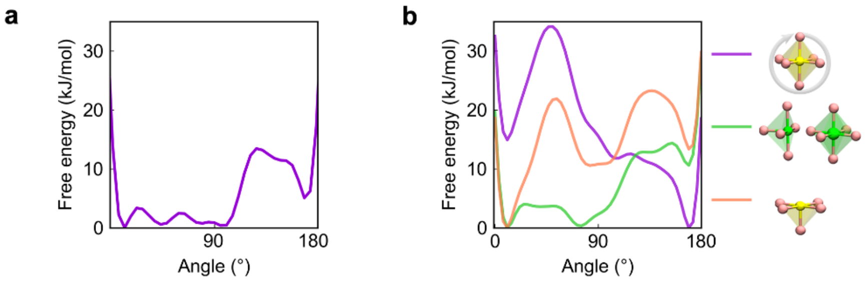

Supplementary Figure 31. Free energy profiles of intra-LCS lithium-ion diffusion. a-b, Free energy plot of intra-LCS lithium-ion diffusion in LPSCl-II and LPSCl-III, respectively. The angle is between the Li-S bond and the z-axis.

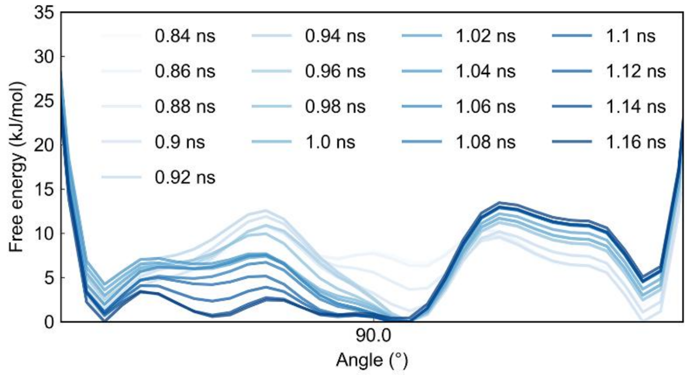

Supplementary Figure 32. Free energy convergence of intra-LCS lithium-ion diffusion in LPSCl-II from 0.84 ns to 1.16 ns.

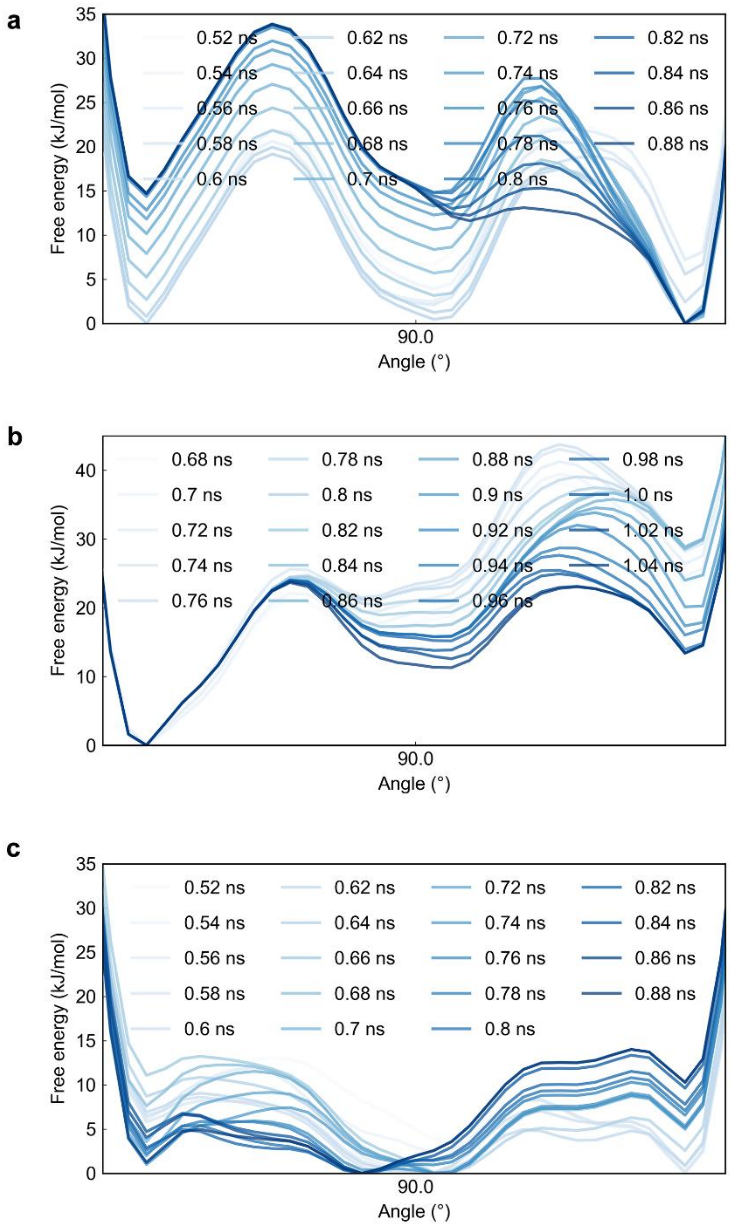

Supplementary Figure 33. Free energy convergence of intra-LCS lithium-ion diffusion in LPSCl-III. a, Free energy convergence of the intra-LCS lithium-ion diffusion in S-centered LCS from 0.52 ns to 0.88 ns. **b,** Free energy convergence of the intra-LCS lithium-ion diffusion in Cl-centered LCS from 0.68 ns to 1.04 ns. **c**, Free energy convergence of the intra-LCS lithium-ion diffusion in S-centered LCS with one lithium vacancy from 0.52 ns to 0.8 ns.

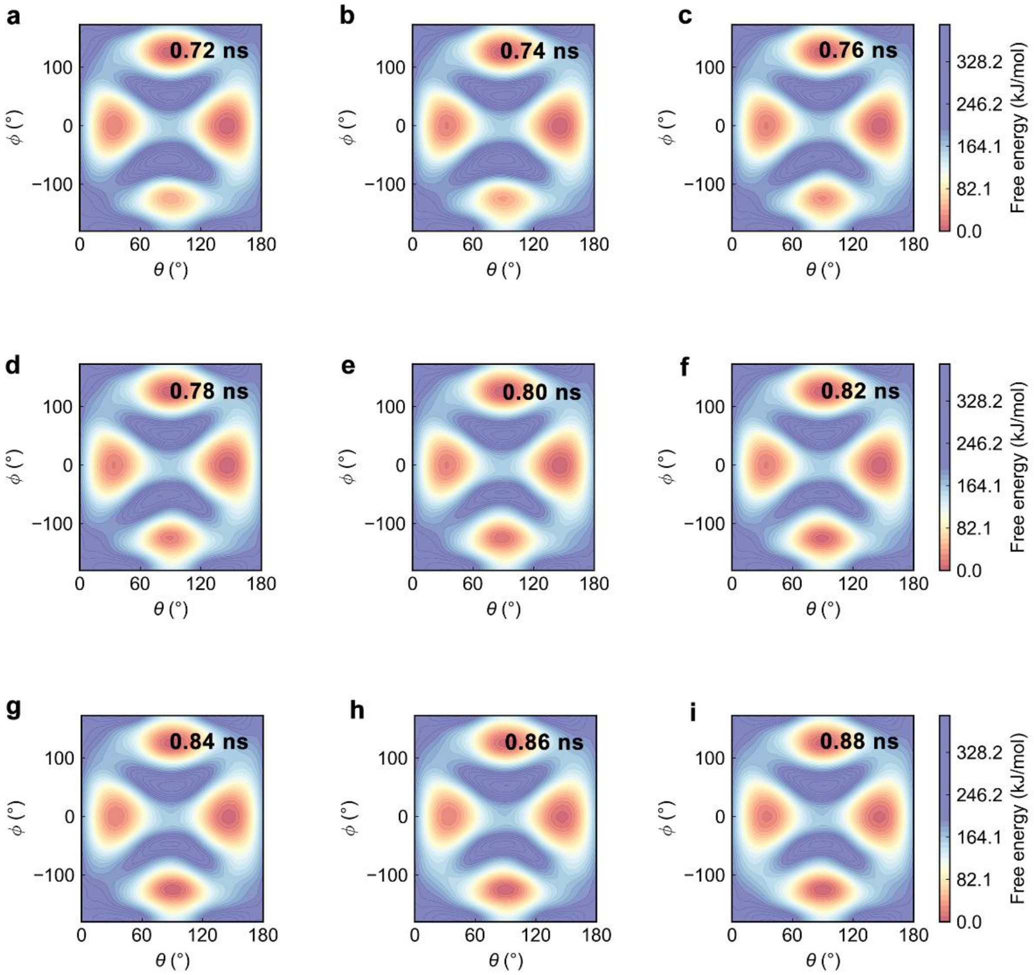

Supplementary Figure 34. Rotational free energy convergence of $[PS_4]^{3-}$ at 300 K in LPSCl-I from 0.72 ns to 0.88 ns.

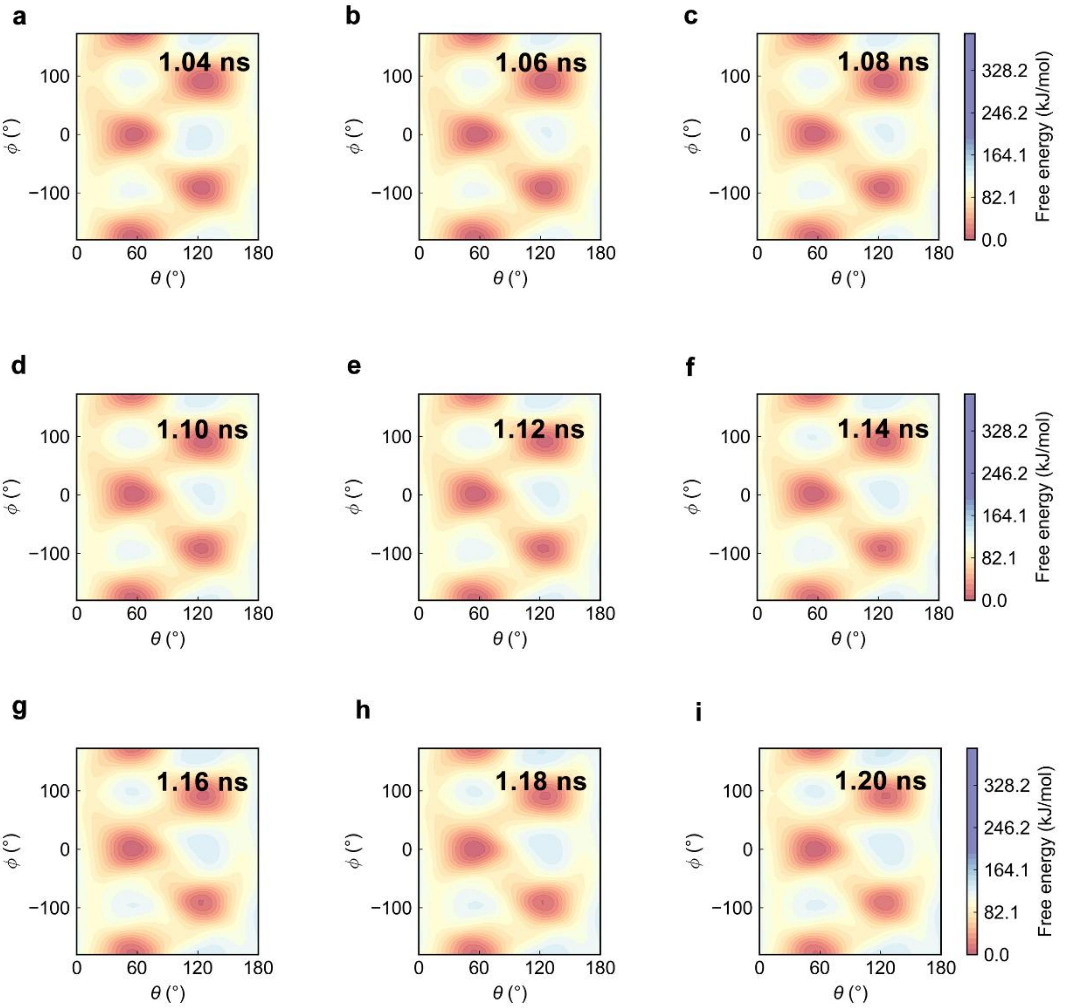

Supplementary Figure 35. Rotational free energy convergence of $[PS_4]^{3-}$ at 300 K in LPSCl-II from 1.04 ns to 1.20 ns.

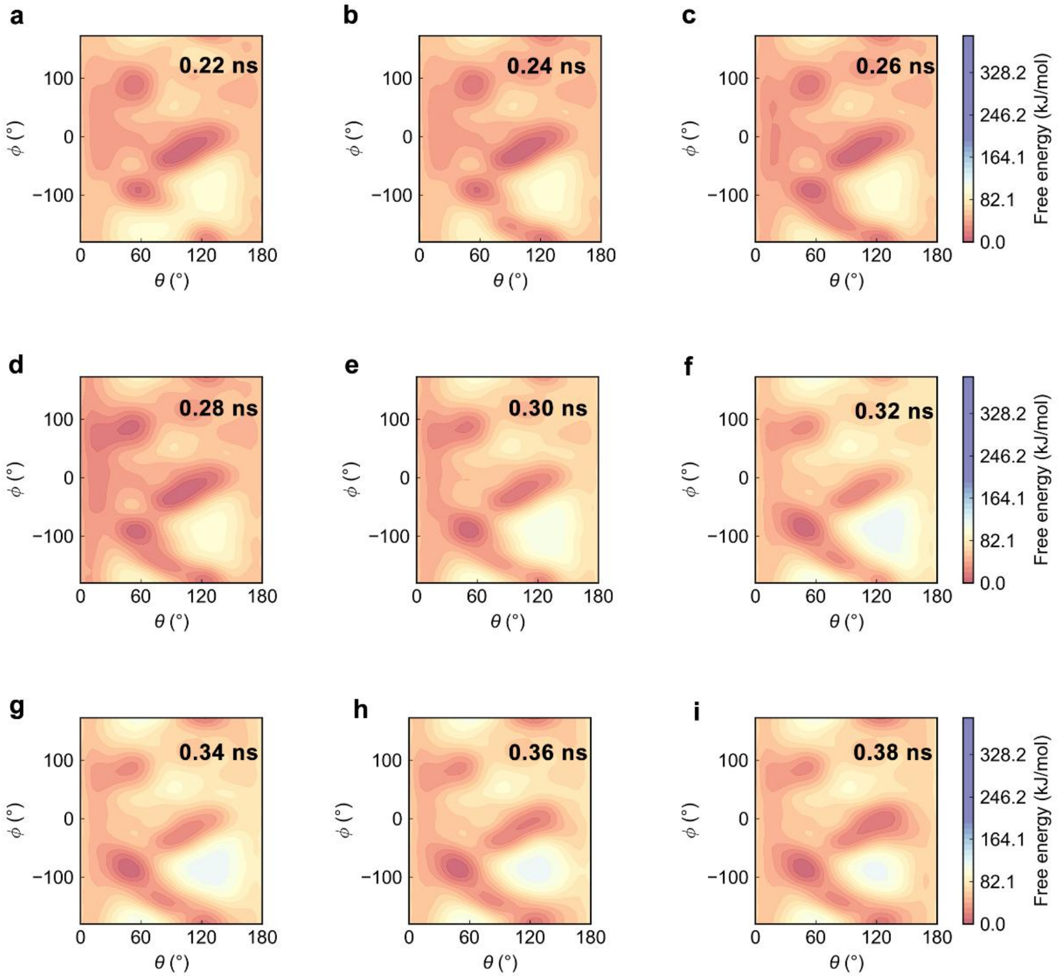

Supplementary Figure 36. Rotational free energy convergence of $[PS_4]^{3-}$ at 300 K in LPSCl-III from 0.22 ns to 0.38 ns.

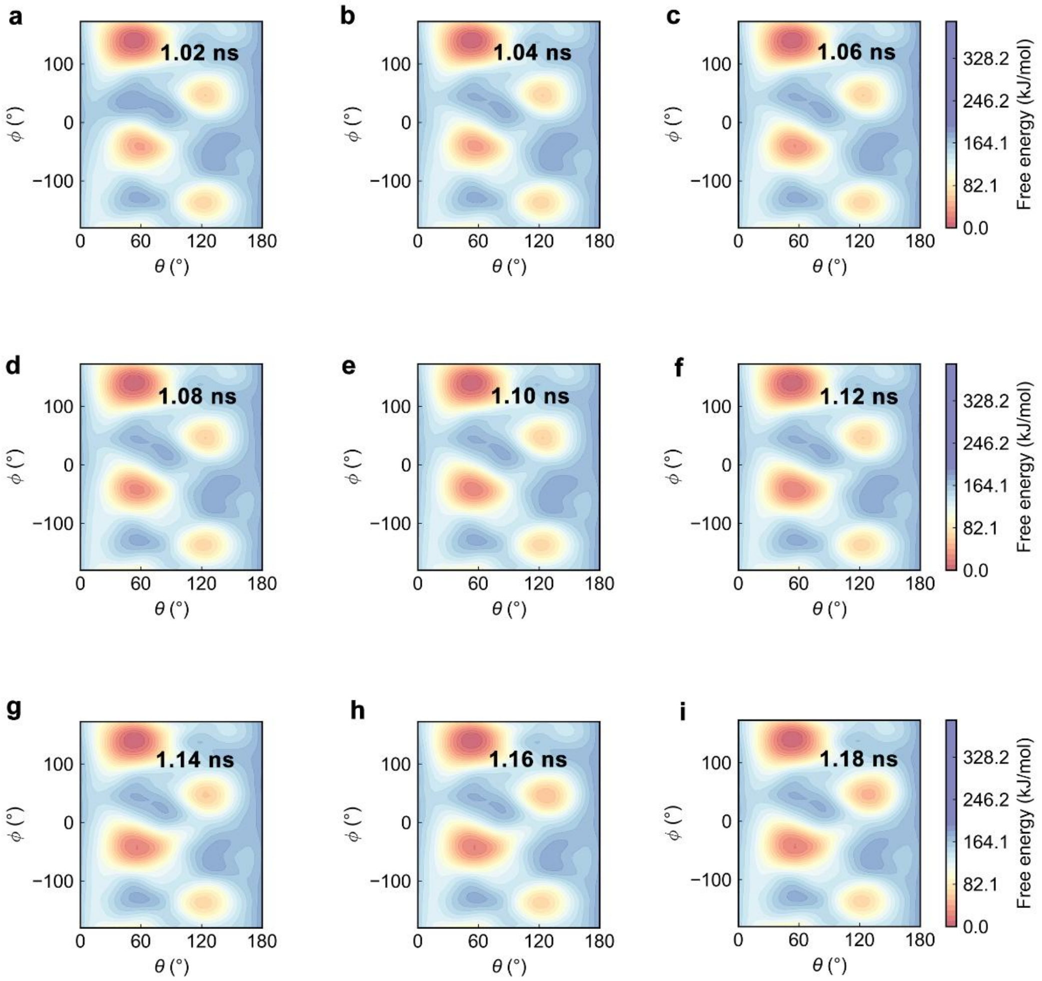

Supplementary Figure 37. Rotational free energy convergence of $[PS_4]^{3-}$ at 300 K in LSPSCl from 1.02 ns to 1.18 ns.

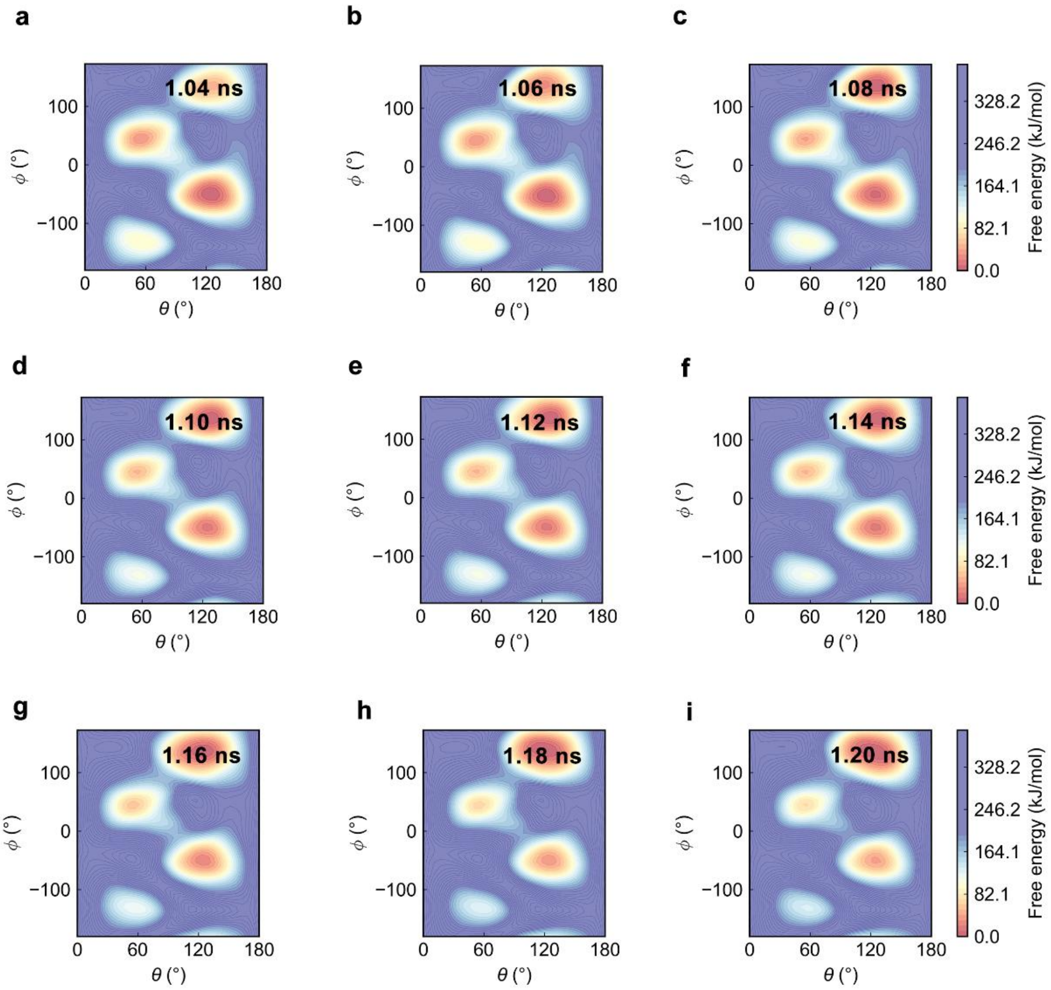

183

184 **Supplementary Figure 38.** Rotational free energy convergence of $[SiS_4]^{4-}$ at 300 K in
185 LSPSCl from 1.04 ns to 1.20 ns.

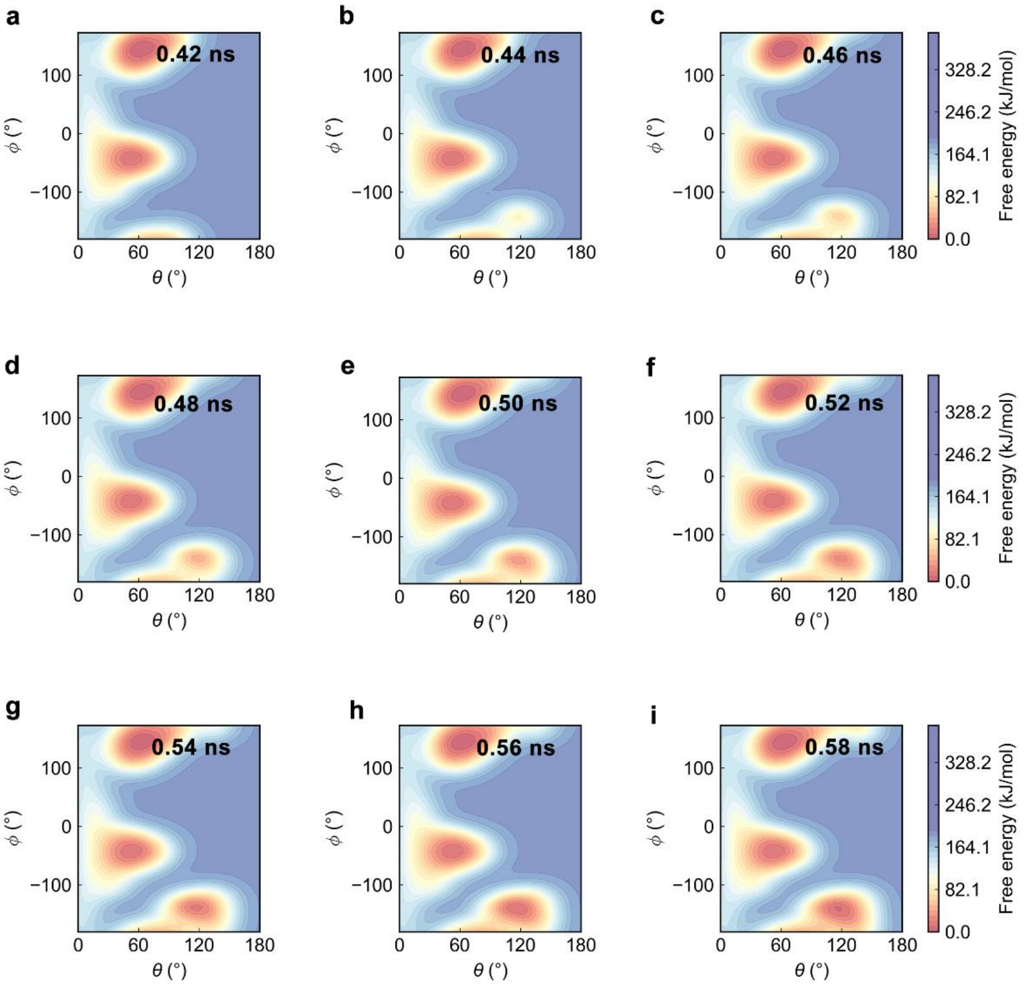

Supplementary Figure 39. Rotational free energy convergence of $[SiS_3Cl]^{3-}$ at 300 K in LSPSCl from 0.42 ns to 0.58 ns.

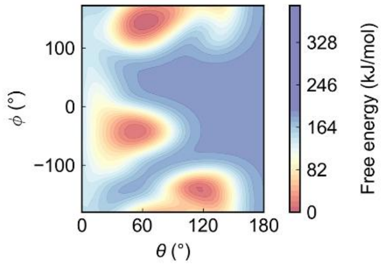

Supplementary Figure 40. Rotational free energy profile of the $[SiS_3Cl]^{3-}$ units in LSPSCl.

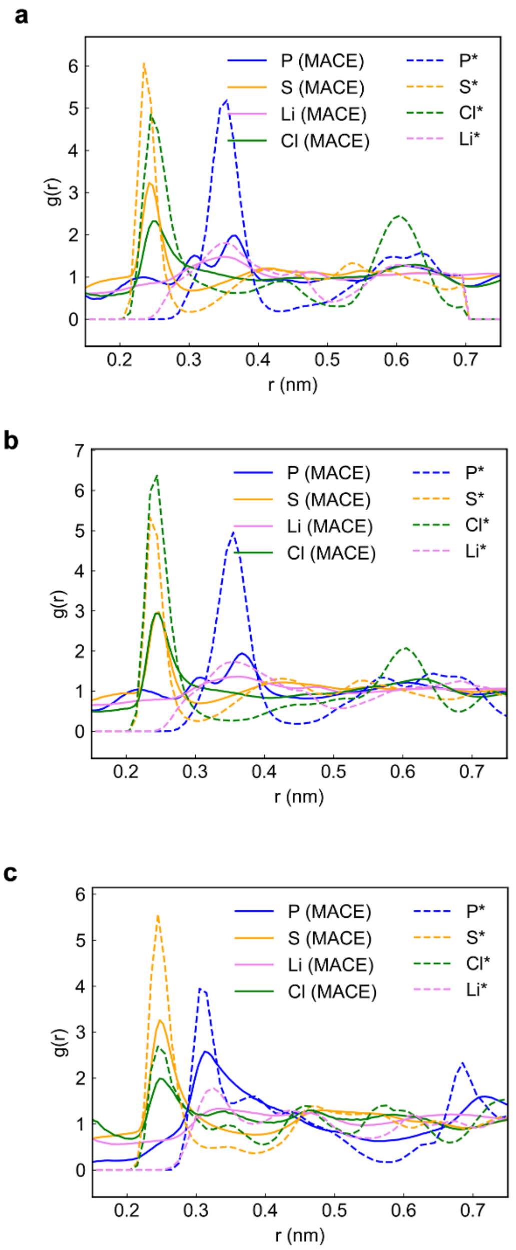

Supplementary Figure 41. Radial distribution analysis of MACE potential. Radial distribution function plot of lithium with P, S, Li, and Cl elements at 300 K under NVT ensemble from MACE potential[10] for **a**, LPSCl-II, **b**, LSPSCl, and **c**, LPSCl-III. The dotted and solid lines represent the calculation from DFT and MACE potential, respectively.

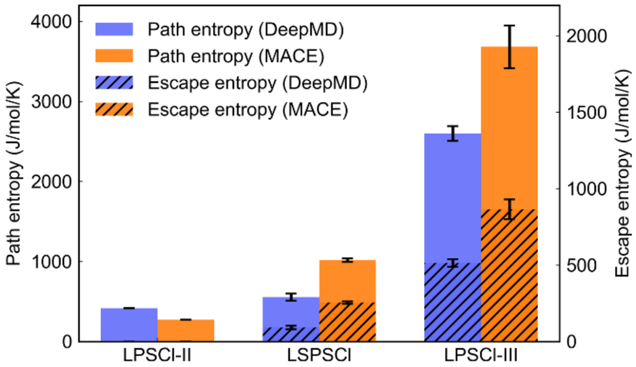

Supplementary Figure 42. Path entropy comparison of NNMD results from DeepMD and MACE potentials. Data are presented as mean values, error bars denote the 95% confidence interval of path entropy calculated from three transfer probability cutoffs of 0.14, 0.15, and 0.16.

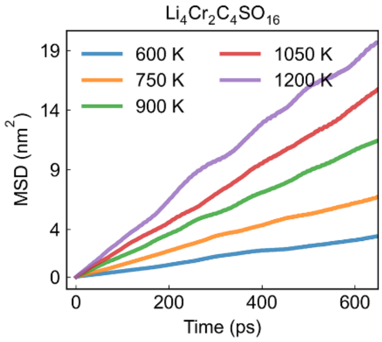

Supplementary Figure 43. Mean squared displacements (MSDs) of $Li_4Cr_2C_4SO_{16}$.

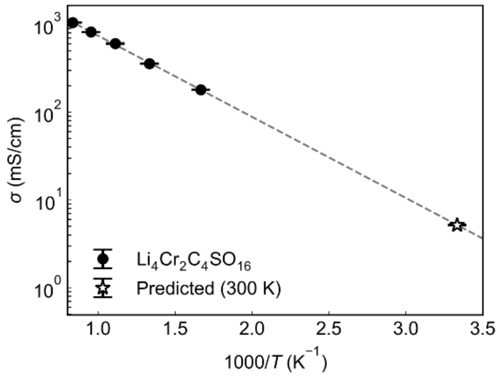

Supplementary Figure 44. Arrhenius plot of ionic conductivities of $Li_4Cr_2C_4SO_{16}$. The corresponding activation energy is 0.18 eV. The uncertainty was estimated using

block averaging with N = 5 independent time origins. Data are presented as mean values, with error bars denoting the 95% confidence interval.

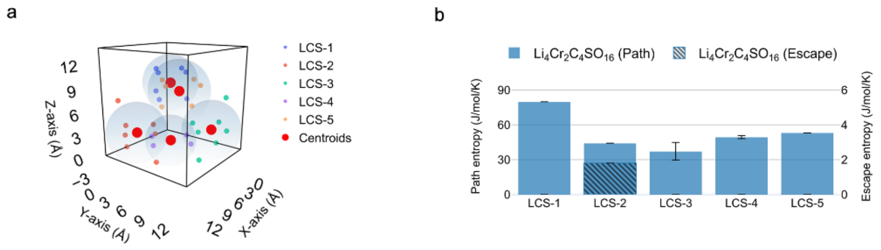

Supplementary Figure 45. Distribution of LCSs and path entropies in $Li_4Cr_2C_4SO_{16}$. **a**, distribution of LCSs in $Li_4Cr_2C_4SO_{16}$. **b**, bar plot of LCS-based path entropy and escape entropy. Path entropies are presented as mean values over three independent trajectories, each evaluated at three transfer probability cutoffs of 0.14, 0.15, and 0.16. Error bars denote the 95% confidence interval.

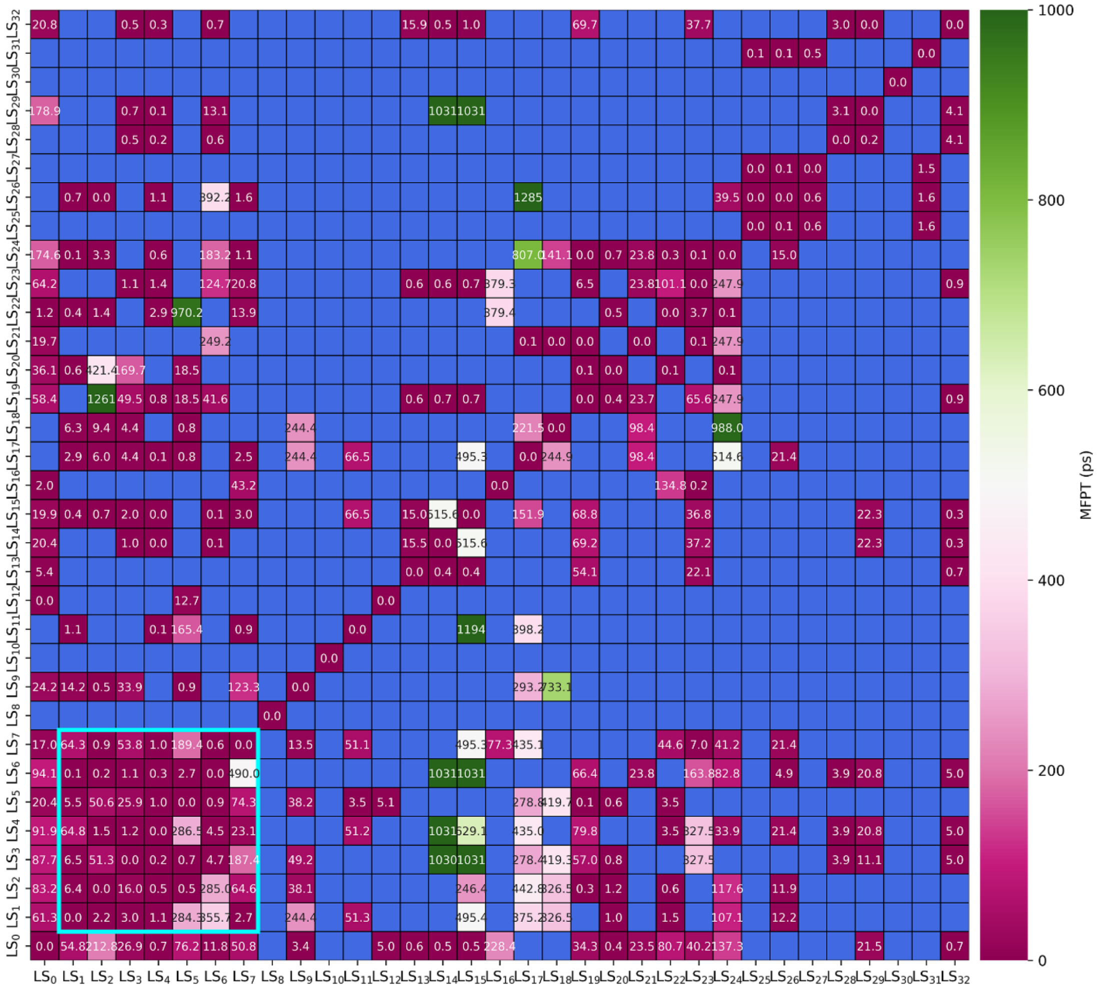

Supplementary Figure 46. MFPT profile of lithium hopping in $Li_4Cr_2C_4SO_{16}$ at 300 K of LCS-1. The initial lithium states in LCS-1 are denoted with a cyan rectangle. Blocks colored blue represent no transition happens or have MFPT value greater than 10000 ps.

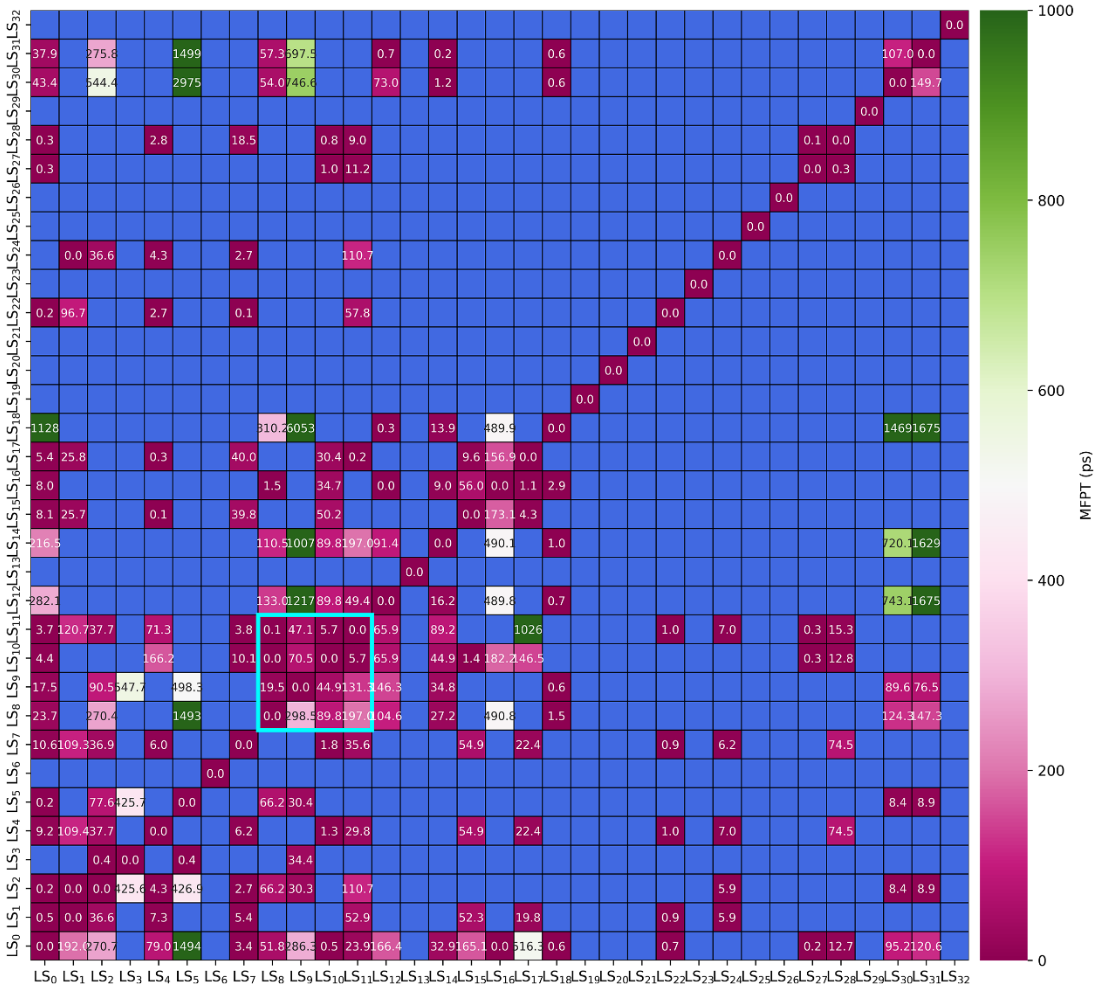

Supplementary Figure 47. MFPT profile of lithium hopping in $Li_4Cr_2C_4SO_{16}$ at 300 K of LCS-2. The initial lithium states in LCS-2 are denoted with a cyan rectangle. Blocks colored blue represent no transition happens or have MFPT value greater than 10000 ps.

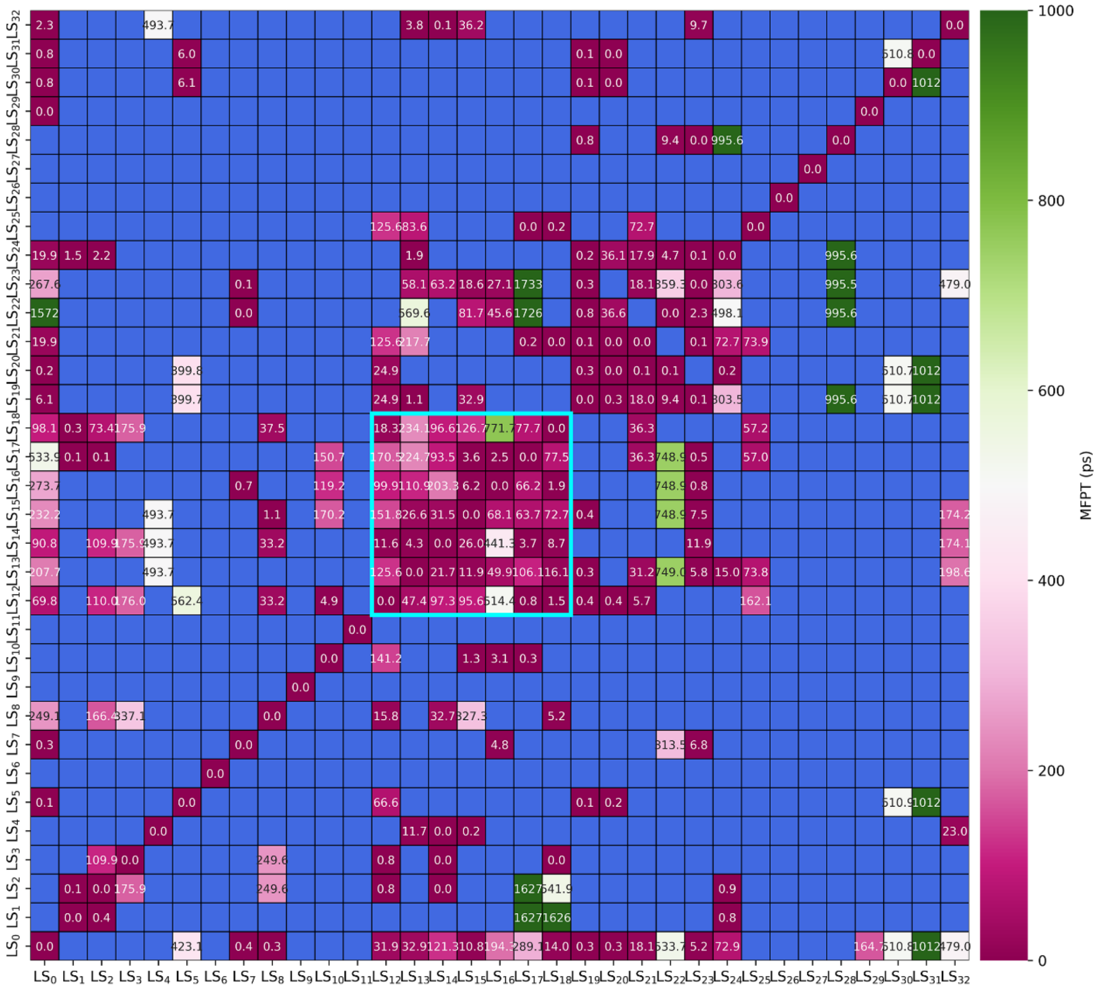

Supplementary Figure 48. MFPT profile of lithium hopping in $Li_4Cr_2C_4SO_{16}$ at 300 K of LCS-3. The initial lithium states in LCS-3 are denoted with a cyan rectangle. Blocks colored blue represent no transition happens or have MFPT value greater than 10000 ps.

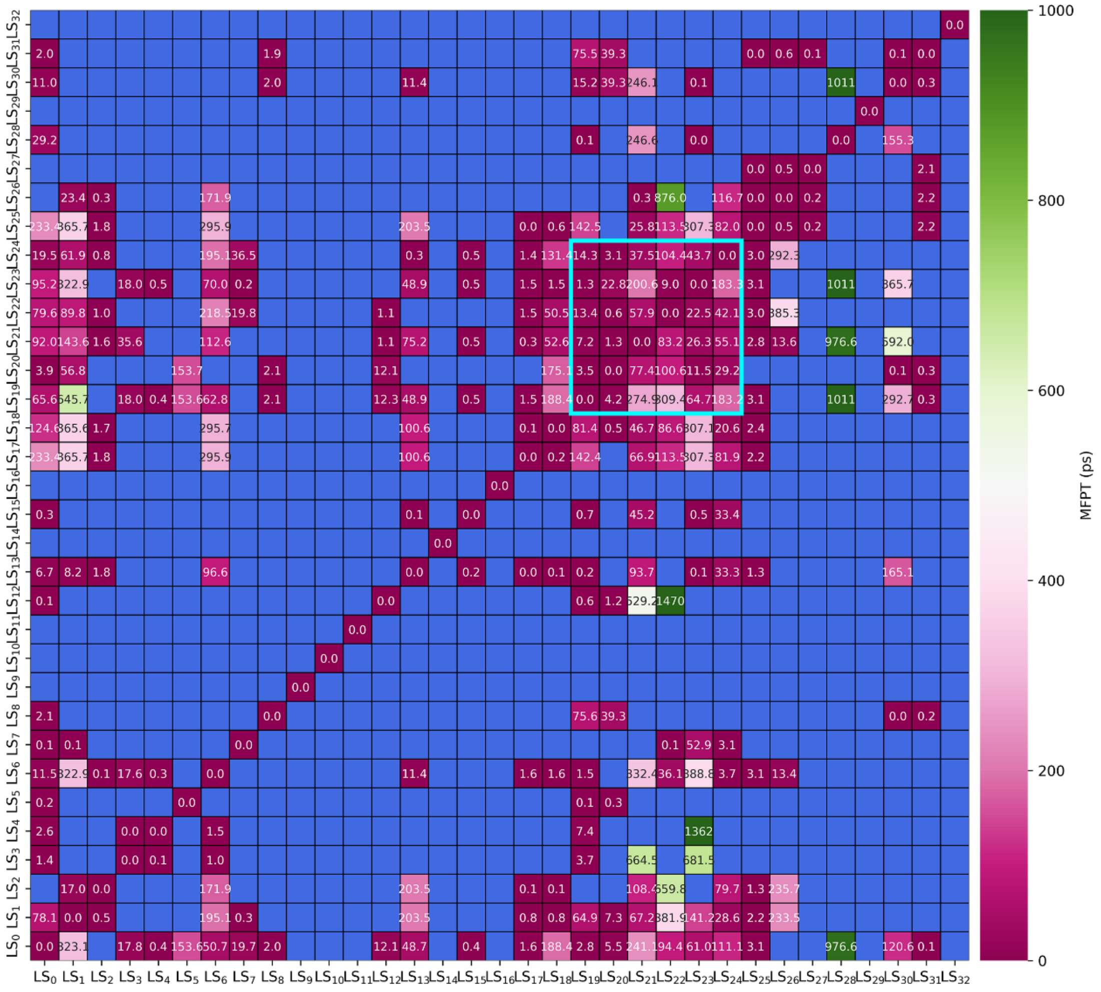

Supplementary Figure 49. MFPT profile of lithium hopping in $Li_4Cr_2C_4SO_{16}$ at 300 K of LCS-4. The initial lithium states in LCS-4 are denoted with a cyan rectangle. Blocks colored blue represent no transition happens or have MFPT value greater than 10000 ps.

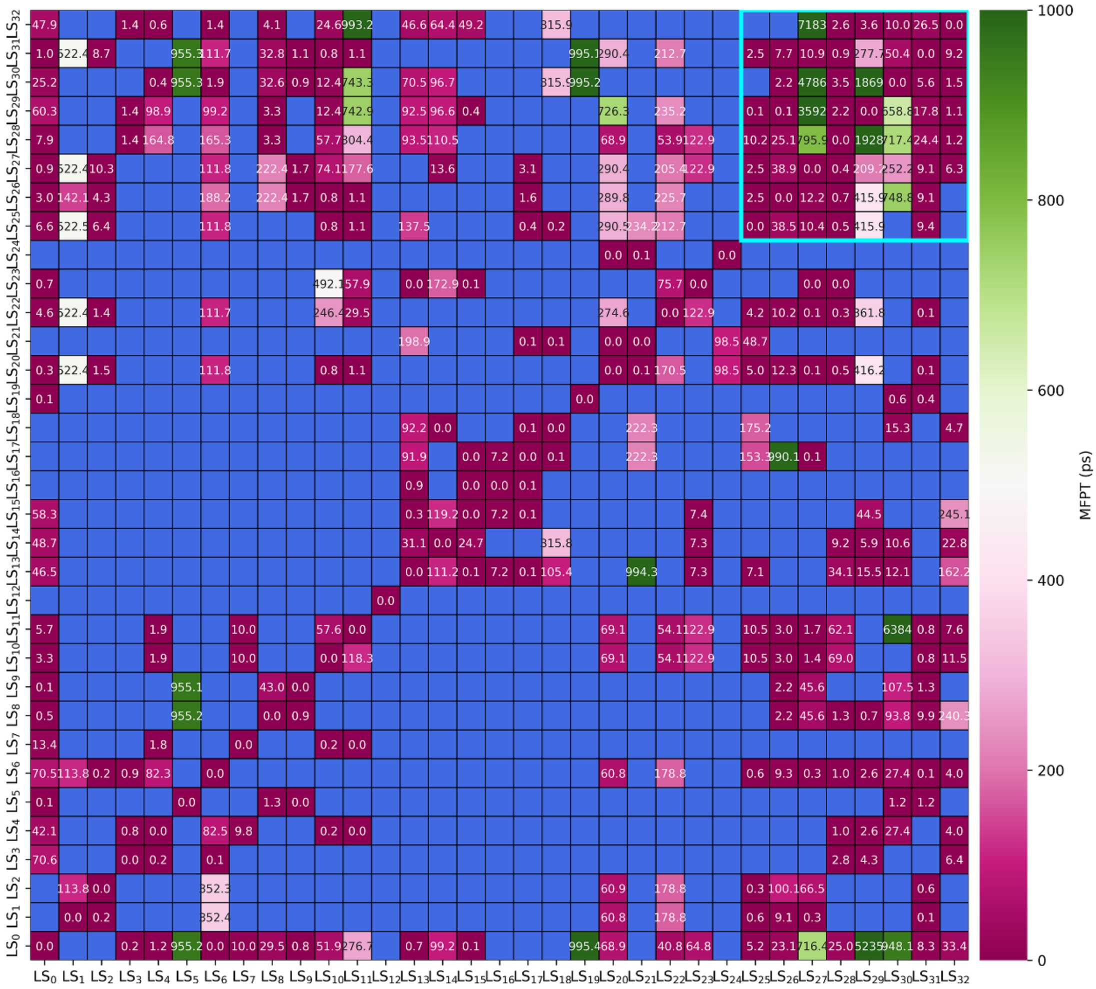

Supplementary Figure 50. MFPT profile of lithium hopping in $Li_4Cr_2C_4SO_{16}$ at 300 K of LCS-5. The initial lithium states in LCS-5 are denoted with a cyan rectangle. Blocks colored blue represent no transition happens or have MFPT value greater than 10000 ps.

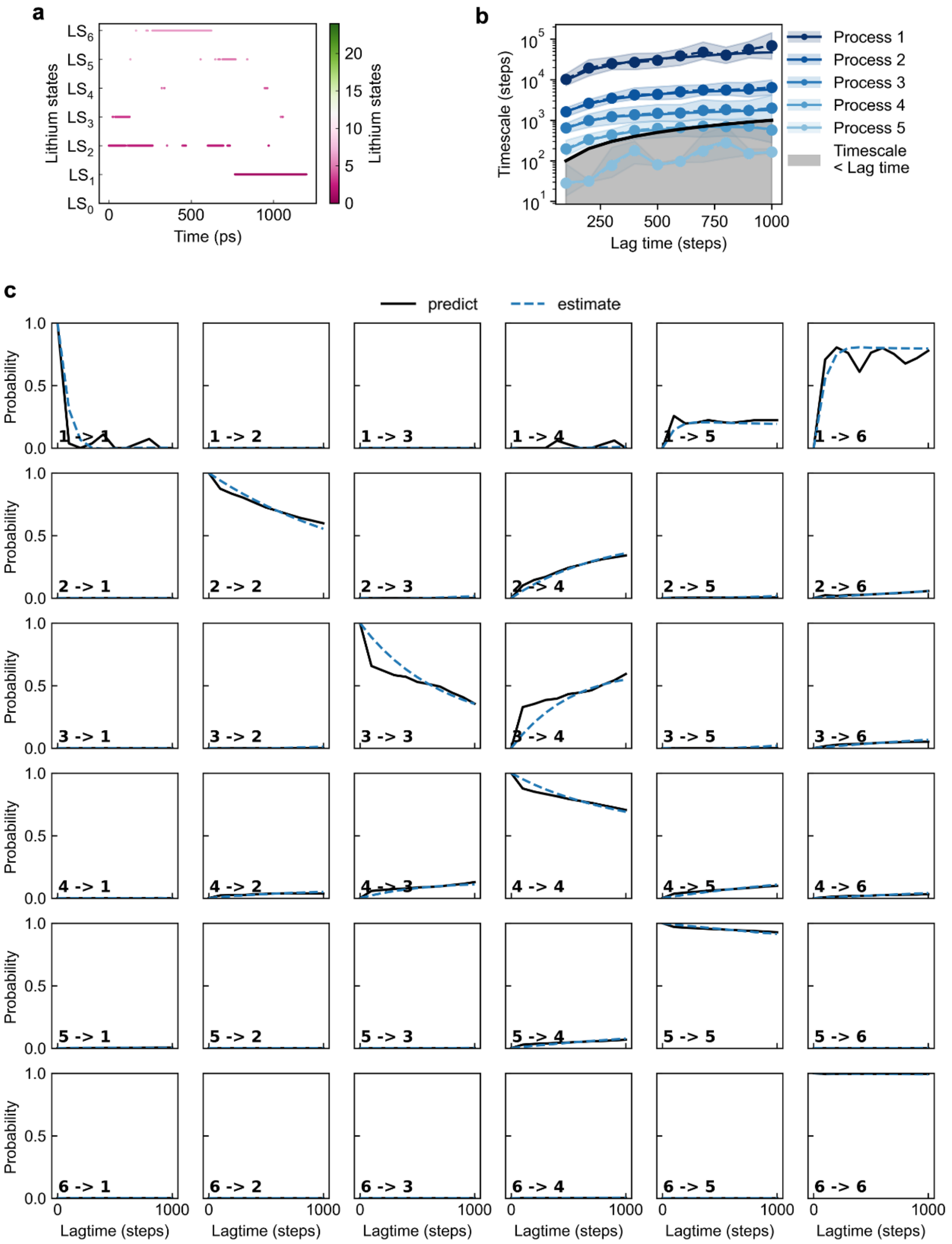

Supplementary Figure 51. Chapman-Kolmogorov tests for one lithium ion in LPSCl-II. a, Time evolution trajectory of a single lithium ion over 1.2 ns (20 fs time resolution) through various lithium states within LPSCl-II. **b**, Implied time scale plot of Markov process in LPSCl-II. The shaded area around each process represents 95%

confidence interval. Region below the solid black line indicates processes occurring faster than the lag time. **c**, Chapman-Kolmogorov test for the 6 most populated microstates.

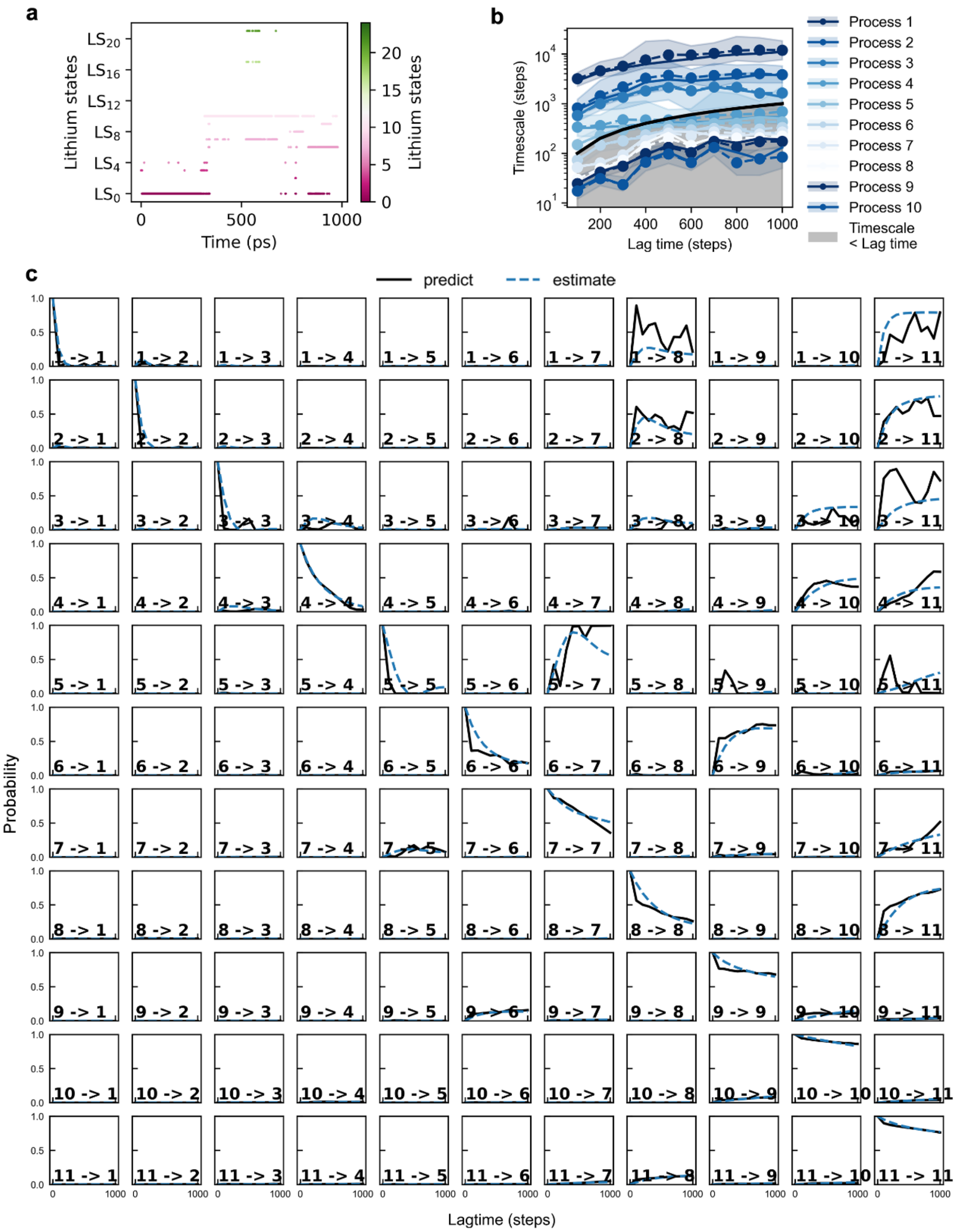

Supplementary Figure 52. Chapman-Kolmogorov tests for one lithium ion in LPSCl-III. a, Time evolution trajectory of a single lithium ion over 1.0 ns (20 fs time

resolution) through various lithium states within LPSCl-III. **b**, Implied time scale plot of Markov process in LPSCl-III. The shaded area around each process represents 95% confidence interval. Region below the solid black line indicates processes occurring faster than the lag time. **c**, Chapman-Kolmogorov test for the 11 most populated microstates.

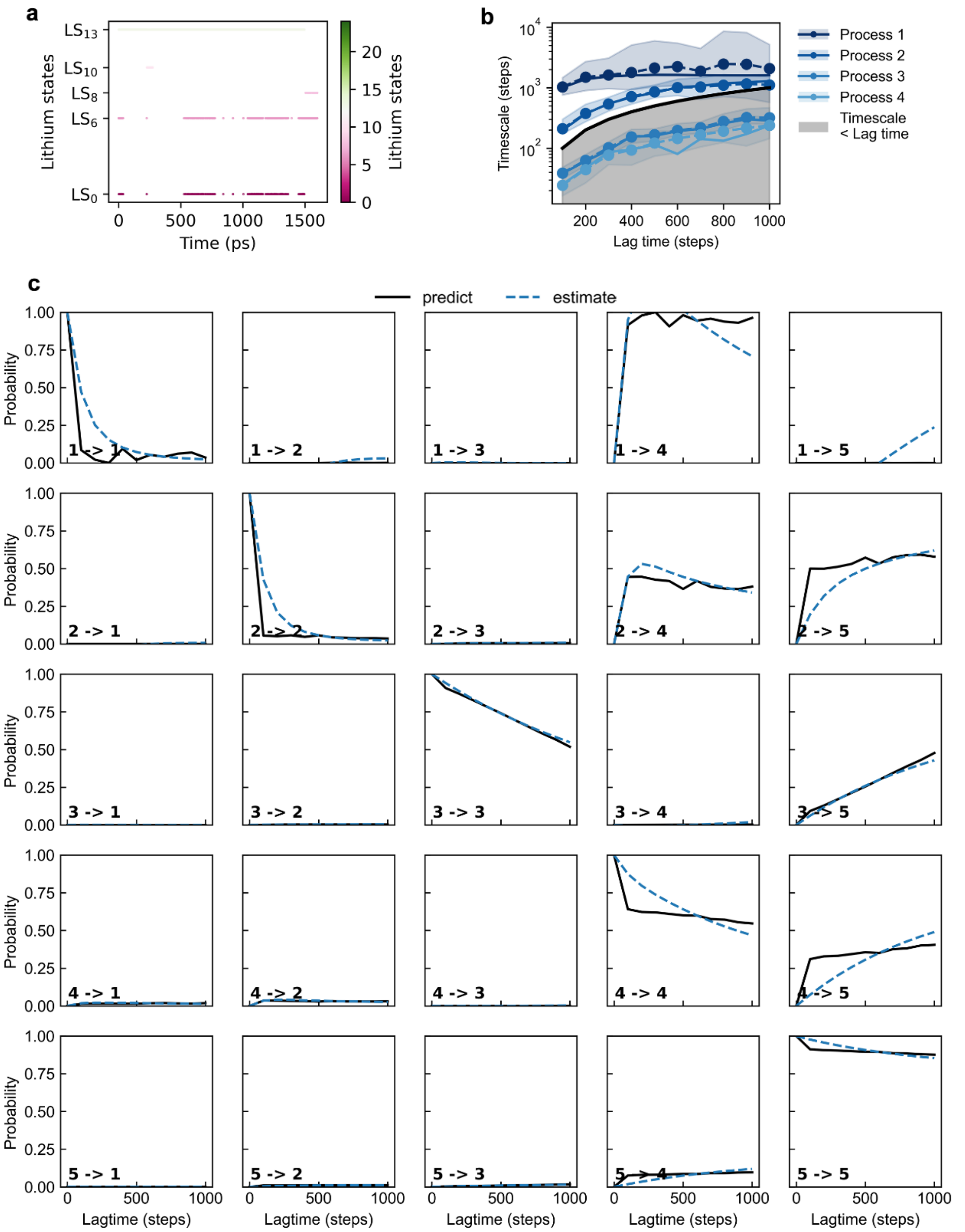

Supplementary Figure 53. Chapman-Kolmogorov tests for one lithium ion in LSPSCl. a, Time evolution trajectory of a single lithium ion over 1.5 ns (20 fs time resolution) through various lithium states within LSPSCl. The shaded area around each process represents 95% confidence interval. Region below the solid black line indicates processes occurring faster than the lag time. **b**, Implied time scale plot of Markov process in LSPSCl. **c**, Chapman-Kolmogorov test for the 5 most populated microstates.

Supplementary Table 1. Diffusion coefficients of LPSCl-I, LPSCl-II, LPSCl-III, and LSPSCl calculated under different temperatures. (unit: m^2/s)

SSE type	600 K	750 K	900K	1050 K	1200 K
LPSCl-I	1.43e-12 ± 9.38e-14	1.43e-12 ± 9.38e-14	1.43e-12 ± 9.38e-14	1.43e-12 ± 9.38e-14	1.43e-12 ± 9.38e-14
LPSCl-II	3.30e-11 ± 5.56e-13	3.30e-11 ± 5.56e-13	3.30e-11 ± 5.56e-13	3.30e-11 ± 5.56e-13	3.30e-11 ± 5.56e-13
LPSCl-III	1.09e-09 ± 1.27e-12	1.09e-09 ± 1.27e-12	1.09e-09 ± 1.27e-12	1.09e-09 ± 1.27e-12	1.09e-09 ± 1.27e-12
LSPSCl	2.79e-10 ± 2.31e-12	2.79e-10 ± 2.31e-12	2.79e-10 ± 2.31e-12	2.79e-10 ± 2.31e-12	2.79e-10 ± 2.31e-12

Supplementary Table 2. The parameters for training neural network potentials (NNPs).

SSE type	Cutoff (Å)	Hidden layers	Fitting layers	Learning rate	Decay rate	Energy pre-factor	Force pre-factor
LPSCl-I	8.5	25, 50, 100	240, 240, 240	0.001	2000	0.02	1000
LPSCl-II	8.5	25, 50, 100	240, 240, 240	0.001	2000	0.02	1000
LPSCl-III	6.5	25, 50, 100	480, 480, 480	0.001	2000	0.02	1000
LSPSCl	6.5	25, 50, 100	480, 480, 480	0.001	2000	0.02	2000

Supplementary Table 3. Mean absolute errors (MAEs) of NNPs.

MAE	LPSCl-I	LPSCl-II	LPSCl-III	LSPSCl
Energy MAE (meV/atom)	1.11 ± 0.02	1.15 ± 0.03	0.58 ± 0.02	0.96 ± 0.02
Force MAE (meV/Å)	38.15 ± 0.35	27.47 ± 0.08	37.07 ± 0.66	42.27 ± 0.34

Supplementary Table 4. Path entropy S_p (J/mol/K) values of LCSs in LPSCl-I, LPSCl-II, LPSCl-III, and LSPSCl.

**LCS/SSE type**	**LPSCl-I**	**LPSCl-II**	**LPSCl-III**	**LSPSCl**
LCS-1	0.00	108.47 ± 1.10	138.57 ± 9.47	235.71 ± 16.48
LCS-2	0.00	102.32 ± 1.75	1018.32 ± 34.68	76.58 ± 5.06
LCS-3	0.00	102.19 ± 1.82	149.51 ± 14.87	169.68 ± 20.09
LCS-4	-	102.74 ± 1.35	1291.76 ± 53.57	53.08 ± 7.71
LCS-5	-	-	-	235.71 ± 16.48
Total	0.00	415.72 ± 6.02	2598.16 ± 112.58	553.91 ± 54.42

Supplementary Table 5. Escape entropy S_e (J/mol/K) values of LCSs in LPSCl-I, LPSCl-II, LPSCl-III and LSPSCl.

**LCS**	**LPSCl-I**	**LPSCl-II**	**LPSCl-III**	**LSPSCl**
LCS-1	0.00	0.00	0.00 ± 0.00	37.34 ± 4.43
LCS-2	0.00	0.00	162.89 ± 7.69	2.42 ± 1.46
LCS-3	0.00	0.00	0.00 ± 0.00	46.81 ± 6.87
LCS-4	-	0.00	350.55 ± 19.17	4.59 ± 2.56
LCS-5	-	-	-	37.34 ± 4.43
Total	0.00	0.00	513.44 ± 26.86	91.70 ± 15.82

Supplementary Table 6. Effects of cutoff values from Voronoi partitioning on path entropy S_p .

SSE type/Cutoff	1.5 Å	1.75 Å	2.00 Å
**LPSCl-I**	0.00	0.00	0.00
**LPSCl-II**	486.99 ± 12.51	415.72 ± 6.02	414.35 ± 4.87
**LPSCl-III**	2401.02 ± 118.17	2598.16 ± 112.58	2717.91 ± 97.91
**LSPSCl**	530.02 ± 45.69	553.91 ± 54.42	505.78 ± 58.96

Supplementary Table 7. Effects of cutoff values from Voronoi partitioning on escape entropy S_e .

SSE type/Cutoff	1.5 Å	1.75 Å	2.00 Å
**LPSCl-I**	0.00	0.00	0.00
**LPSCl-II**	0.00 ± 0.00	0.00 ± 0.00	0.00 ± 0.00
**LPSCl-III**	492.31 ± 17.52	513.44 ± 26.86	505.83 ± 26.18
**LSPSCl**	86.02 ± 16.44	91.70 ± 15.82	102.00 ± 23.94

Supplementary Table 8. The parameters of rotational free energy calculation of tetrahedron units.

SSE type	Distortion unit	Deposition rate (fs)	Gaussian width	Gaussian height (kJ/mol)	Bias factor
LPSCl-I	$[PS_4]^{3-}$	50	0.25	5	20
LPSCl-II	$[PS_4]^{3-}$	100	0.25	2	10
LPSCl-III	$[PS_4]^{3-}$	100	0.25	2	20
LSPSCl	$[PS_4]^{3-}$	100	0.25	2	20
LSPSCl	$[SiS_4]^{4-}$	50	0.25	2	100
LSPSCl	$[SiS_3Cl]^{3-}$	100	0.25	2	20

Supplementary Table 9. Summary of 27 screened superionic candidates.

No.	Identifier	Space Group	Band gap (eV)	Energy above hull per atom (eV)	Chemical formula	MSD at 300K (nm^2)	Path entropy (J/mol/K)	Escape entropy (J/mol/K)
1	mp-1040451	6	2.39	0.035	$Li_{20}Si_3P_3S_{23}Cl$	0.0118	1016.57 ± 27.10	254.69 ± 10.30
2	mp-532413	9	3.59	0.000	$Li_5B_7S_{13}$	0.0165	629.35 ± 63.38	102.84 ± 22.50
3	agm003282504	105	2.09	0.021	$Li_{10}Ge(PS_6)_2$	0.0156	381.37 ± 51.93	76.60 ± 19.55
4	mp-641703	2	2.49	0.020	$Li_7P_3S_{11}$	0.0178	314.62 ± 18.74	50.73 ± 1.43
5	mp-696123	105	2.27	0.035	$Li_{10}Sn(PS_6)_2$	0.0156	300.41 ± 17.32	46.25 ± 1.64
6	mp-769048	62	2.15	0.078	Li_3NbS_4	0.0117	298.29 ± 15.32	46.02 ± 6.44
7	mp-985592	216	2.30	0.083	Li_6PS_5Cl	0.0243	273.52 ± 0.00	0.00 ± 0.00
8	mp-771912	70	2.30	0.070	$Li_4Cr_2C_4SO_{16}$	0.0141	263.98 ± 10.42	1.80 ± 0.00
9	mp-985591	216	2.14	0.051	Li_6PS_5Br	0.0162	253.08 ± 0.00	0.00 ± 0.00
10	mp-1211324	33	2.08	0.033	Li_7PS_6	0.0119	224.56 ± 42.84	20.67 ± 5.66
11	mp-768991	70	2.05	0.076	$Li_4Bi_2C_4SO_{16}$	0.0128	189.82 ± 25.98	1.51 ± 0.00
12	agm003282544	105	2.41	-0.008	$Li_{10}Si(PS_6)_2$	0.0186	167.51 ± 16.24	17.94 ± 7.20
13	mp-766600	218	2.42	0.069	Li_4TiS_4	0.0196	166.70 ± 18.89	3.92 ± 2.97
14	agm003232549	141	2.02	0.077	Li_3CuS_2	0.0165	95.80 ± 12.33	1.76 ± 1.64
15	mp-1222582	33	2.51	0.026	Li_4GeS_4	0.0220	65.66 ± 0.89	0.70 ± 0.14
16	mp-766575	33	2.70	0.063	Li_8TiS_6	0.0184	50.37 ± 24.03	0.93 ± 1.82
17	agm003230254	7	4.74	0.041	$LiH_3(SeO_3)_2$	0.0251	22.39 ± 0.06	0.00 ± 0.00
18	mp-849740	1	2.08	0.028	$LiFe(SO_4)_2$	0.0135	17.78 ± 0.00	1.41 ± 0.00
19	agm003235290	62	4.39	0.064	$CsLiSO_4$	0.0122	16.33 ± 0.00	0.00 ± 0.00
20	agm002219141	82	2.01	0.030	$LiCdPSe_4$	0.0246	4.14 ± 0.00	0.00 ± 0.00
21	mp-769554	1	2.43	0.009	$LiMgCr_3(SO_4)_6$	0.0241	0.00 ± 0.00	0.00 ± 0.00

22	mp-754902	4	4.06	0.068	$LiSbCSO_7$	0.0220	0.00 ± 0.00	0.00 ± 0.00
23	mp-695469	1	2.25	0.028	$Li_3Cr_{13}Ni_3(SO_4)_{24}$	0.0202	0.00 ± 0.00	0.00 ± 0.00
24	mp-769549	1	2.32	0.011	$LiZnCr_3(SO_4)_6$	0.0193	0.00 ± 0.00	0.00 ± 0.00
25	mp-770759	4	3.75	0.070	$LiBiCSO_7$	0.0174	0.00 ± 0.00	0.00 ± 0.00
26	mp-766088	1	2.33	0.009	$LiCr_3Ni(SO_4)_6$	0.0179	0.00 ± 0.00	0.00 ± 0.00
27	agm002342398	164	2.04	0.072	LiS_2Sm	0.0124	0.00 ± 0.00	0.00 ± 0.00

Supplementary Table 10. Diffusion coefficient of $Li_4Cr_2C_4SO_{16}$ calculated under different temperatures. (unit: m^2/s)

SSE type	600 K	750 K	900K	1050 K	1200 K
$Li_4Cr_2C_4SO_{16}$	8.73e-10 ± 6.35e-12	1.73e-09 ± 1.28e-11	2.93e-09 ± 3.98e-11	3.97e-09 ± 1.38e-11	5.07e-09 ± 2.46e-11

1. Supplementary Note 1

Calculation of lithium-ion conductivity.

To get ionic conductivities at room temperature, we performed a wide range neural network potential-based molecular dynamics (NNMD) simulations under 600 K to 1200 K (600 K, 750 K, 900 K, 1050 K, and 1200K). For LPSCl-I, a supercell with 4608 atoms (4.71 nm × 41.46 nm × 42.83 nm) was used. For LPSCl-II, a supercell with 3744 atoms (4.29 nm × 4.29nm × 4.04 nm) was used. For LPSCl-III, a supercell with 3600 atoms (4.36 nm × 4.36 nm × 4.11 nm) was used. For LSPSCl, a supercell with 3600 atoms (3.69 nm × 3.69 nm × 5.17 nm) was used. Prior to analysis, the first 100 ps of relaxation at the target temperature was excluded. Then we first computed the diffusion coefficient (D) using the mean square displacement (MSD) of lithium-ions (Supplementary Figure 4):

$$D = \frac{MSD(t)}{2d\Delta t} = \frac{|\ x_i(t) - x_i(t_0)\ |^2 + |\ y_i(t) - y_i(t_0)\ |^2 + |\ z_i(t) - z_i(t_0)\ |^2}{2d\Delta t} \quad (1)$$

After having D at high temperatures (≥ 600 K), we extrapolated D at room temperature 300 K. Finally, the conductivity (σ) is calculated from the Nernst-Einstein relation:

$$\sigma = \frac{Nq^2}{Vk_{\mathrm{B}}T}D \tag{2}$$

Where N is the number of lithium ions, V is the total volume of the simulation cell, q is the charge of the lithium-ion, T is the temperature, and k_{B} is the Boltzmann constant.

2. Supplementary Note 2

2.1. Neural-network potential validation and accuracy.

To validate the neural-network potentials (NNPs), the mean absolute errors (MAEs) of all the models are listed in Supplementary Table 2. We also plotted the comparison between energies and forces calculated from DFT and predicted from our trained models. (Supplementary Figures 7-10) In addition, the radial distribution functions (RDFs) of lithium ions were calculated and compared between the results from DFT and NNP predictions. (Supplementary Figure 11) From these assessments, the trained models are ready to use in the following production.

2.2. NNMD simulation parameters

NNMD simulations used for MSM construction were performed under the NVT ensemble at 300 K through LAMMPS[7] with i-Pi[8] software for at least 1.0 nano second. For LPSCl-I, a supercell with 4608 atoms (4.71 nm × 41.46 nm × 42.83 nm) was used. For LPSCl-II, a supercell with 3744 atoms (4.29 nm × 4.29nm × 4.04 nm) was used. For LPSCl-III, a supercell with 3600 atoms (4.36 nm × 4.36 nm × 4.11 nm) was used. For LSPSCl, a supercell with 3600 atoms (3.69 nm × 3.69 nm × 5.17 nm) was used.

3. Supplementary Note 3

Periodic K-means and Voronoi partition.

To identify local coordination shells (LCSs), we implemented periodic K-means clustering algorithm using the scikit-learn package[11]. The number of LCSs, denoted as K (where K is less than or equal to the total number of lithium atoms in the unit cell), was determined by minimizing the within-cluster sum of squares (WCSS). Following cluster identification, a Voronoi partition including application of periodic boundary conditions was performed in three dimensions for each LCS[12]. For Voronoi partitioning calculations, we defined a 3D box extending 1.75 Å from the maximum/minimum atomic positions of each LCS. Lithium atoms are considered to

transition from an original LCS to a new LCS only when they move from the overlapping region of the original LCS to another LCS. Uncertainty analysis across this cutoff range (1.50–2.00 Å) was systematically evaluated and summarized in **Supplementary Tables 6** and **7**.

4. Supplementary Note 4

4.1. Inter-LCS diffusion

For lithium-ion diffusion across LCSs, collective variables are defined as two distances from the neighboring LCS center. (Supplementary Figure 27) The simulation parameters setup for well-tempered metadynamics (WTmetaD) employs Gaussian "hills" with a deposition rate of 100 fs, width of 0.20 Å, bias factor of 20, and height of 5.0 kJ/mol for LPSCl-III. For LPSCl-II, the deposition rate is set to 50 fs, while other parameters remain unchanged. A pair of walls is applied to limit the D_1 and D_2 between 1.5 Å and 8.5 Å to constrain the lithium-ion diffusion within neighboring LCSs.

4.2. Intra-LCS diffusion

For intra-LCS diffusion of lithium ion in LPSCl-II and LPSCl-III, the CV is designed as the polar angle formed between bond of lithium and the coordinated center atom (sulfur, chlorine) of LCS with unit vector along Z direction (Supplementary Figure 30) as

$$\theta = \cos^{-1}\left(\frac{\mathbf{v}_1 * \mathbf{n}}{|\mathbf{v}_1||\mathbf{n}|}\right) \quad (3)$$

where $\mathbf{v}_1$is the vector of the Li-S (Cl) bond, and $\mathbf{n}$ is the unit vector (0, 0, 1) of the Z axis. The simulation parameters setup for WTmetaD employs Gaussian "hills" with a deposition rate of 100 fs, width of 0.20 Å, bias factor of 10, and height of 2.0 kJ/mol. A pair of walls is applied to limit the $|v_1|$ between 1.5 Å and 3.0 Å to constrain the lithium ion stays within LCS.

4.3. Distortion of tetrahedron units

The distortion of tetrahedron units in all four types of SSEs is explored. To get the exact free energy barrier of the rotation, the azimuthal angles θ and ϕ are selected as the CVs. The detailed simulation parameters are listed in Supplementary Table 8.

5. Supplementary Note 5

Calculation of configurational disorder.

The tetrahedron distortion δ_d is calculated through:

$$\delta_\mathrm{d} = \frac{1}{\sum_j \xi(r_{ij})} \sum_j \xi(r_{ij}) \left[\frac{(x_{ij} + y_{ij} + z_{ij})^3}{r_{ij}^3} + \frac{(-x_{ij} + y_{ij} - z_{ij})^3}{r_{ij}^3} + \frac{(-x_{ij} - y_{ij} + z_{ij})^3}{r_{ij}^3} \right] \quad (4)$$

$$\xi(r_{ij}) = \frac{1 - (r_{ij} - d_0/r_0)^n}{1 - (r_{ij} - d_0/r_0)^m} \quad (5)$$

Where r_{ij} represents the magnitude of the vector connecting atom i to atom j, and x_{ij}, y_{ij}, and z_{ij} are its' three components corresponding to the X, Y, and Z axes. $\xi(r_{ij})$ is a switching function applied to the distance between atoms i and j. We choose 0.0 Å for d_0 and 3.0 Å for r_0. Three independent simulations were performed to compute δ_d and its corresponding configurational entropy. The mean configurational entropy values fall within the 99.5% confidence interval.

6. Supplementary Note 6

6.1. Validation of MACE neural-network potential.

Neural network potential-based molecular dynamics simulations were performed utilizing the MACE pretrained foundational model (medium size)[10]. The accuracy of this model was assessed by comparing radial distribution function (RDF) calculations to analogous results from AIMD, as shown in (Supplementary Figure 41). While the MACE foundational model's accuracy is somewhat lower than that achieved with actively trained DeepMD-kit models (Supplementary Figure 11), it effectively captures the characteristic RDF peaks and demonstrates broad applicability across a wide range of materials, which is essential for high-throughput screening. Furthermore, consistent results for path entropy were obtained using both MACE and DeepMD-kit (Supplementary Figure 42), providing strong validation for the suitability of the MACE foundational model in this high-throughput context.

6.2. MACE foundation model based molecular dynamics.

The MACE foundation model-based molecular dynamics simulations were performed using the ASE software package[13]. A time step of 2.0 fs was employed for materials

lacking hydrogen atoms, while a smaller time step of 0.5 fs was used for systems containing hydrogen to ensure accurate force calculations. The Langevin thermostat was used with coupling constants of 1.0 ps. For the 3rd step screening phase focused on calculating mean squared displacements (MSDs), NNMD simulations were conducted over a total time of 12.0 ps using a 2×2×2 supercell. To ensure the system reaches full equilibrium at room temperature, the first 2.0 ps of simulated trajectories were discarded before MSD analysis. To generate path entropies for the final production (4th screening) run encompassing all 27 candidate materials, NNMD simulations were performed for at least 800.0 ps using larger supercells (typically a 3×3×3 supercell, varying depending on cell size).

6.3. Verification of ionic conductivity.

Lithium-ion conductivity was calculated following the same procedure in **Supplementary Note 4.** All NNMD simulations were simulated using MACE foundation model with a 2×2×2 supercell for 800.0 ps.

Supplementary References

1 Adeli, P. *et al.* Boosting Solid-State Diffusivity and Conductivity in Lithium Superionic Argyrodites by Halide Substitution. *Angew. Chem. Int. Ed.* **58**, 8681-8686 (2019).

2 Barroso-Luque, L. *et al.* smol: A Python package for cluster expansions and beyond. *J. Open Source Softw.* **7**, 4504 (2022).

3 Kresse, G. & Furthmüller, J. Efficient iterative schemes for ab initiototal-energy calculations using a plane-wave basis set. *Phys. Rev. B* **54**, 11169-11186 (1996).

4 Perdew, J. P., Burke, K. & Ernzerhof, M. Generalized Gradient Approximation Made Simple. *Phys. Rev. Lett.* **77**, 3865-3868 (1996).

5 Grimme, S., Antony, J., Ehrlich, S. & Krieg, H. A consistent and accurate ab initio parametrization of density functional dispersion correction (DFT-D) for the 94 elements H-Pu. *J. Chem. Phys.* **132**, 154104 (2010).

6 Zhang, Y. *et al.* DP-GEN: A concurrent learning platform for the generation of reliable deep learning based potential energy models. *Comput. Phys. Commun.* **253**, 107206 (2020).

7 Thompson, A. P. *et al.* LAMMPS - a flexible simulation tool for particle-based materials modeling at the atomic, meso, and continuum scales. *Comput. Phys. Commun.* **271**, 108171 (2022).

8 Kapil, V. *et al.* i-PI 2.0: A universal force engine for advanced molecular simulations. *Comput. Phys. Commun.* **236**, 214-223 (2019).

9 Hoffmann, M. *et al.* Deeptime: a Python library for machine learning dynamical models from time series data. *Mach. learn.: sci. technol.* **3**, 015009 (2022).

10 Batatia, I., Kovacs, D. P., Simm, G., Ortner, C. & Csányi, G. MACE: Higher order equivariant message passing neural networks for fast and accurate force fields. *Adv. Neural Inf. Process. Syst.* **35**, 11423-11436 (2022).

11 Pedregosa, F. *et al.* Scikit-learn: Machine learning in Python. *the Journal of machine Learning research* **12**, 2825-2830 (2011).

12 Aurenhammer, F. Voronoi diagrams—a survey of a fundamental geometric data structure. *ACM Comput. Surv.* **23**, 345-405 (1991).

13 Hjorth Larsen, A. *et al.* The atomic simulation environment-a Python library for working with atoms. *J. Phys. Condens. Matter.* **29**, 273002 (2017).
